# Tissue-Specific Floral Transcriptome Analysis of the Sexually Deceptive Orchid *Chiloglottis trapeziformis* Provides Insights into the Biosynthesis and Regulation of Its Unique UV-B Dependent Floral Volatile, Chiloglottone 1

**DOI:** 10.3389/fpls.2017.01260

**Published:** 2017-07-19

**Authors:** Darren C. J. Wong, Ranamalie Amarasinghe, Claudia Rodriguez-Delgado, Rodney Eyles, Eran Pichersky, Rod Peakall

**Affiliations:** ^1^Ecology and Evolution, Research School of Biology, The Australian National University, Canberra ACT, Australia; ^2^Department of Molecular, Cellular, and Developmental Biology, University of Michigan, Ann Arbor MI, United States

**Keywords:** *Chiloglottis trapeziformis*, chiloglottone, semiochemical, sexual deception, UV-B, transcriptome, gene duplication, gene co-expression network

## Abstract

The Australian sexually deceptive orchid, *Chiloglottis trapeziformis*, employs a unique UV-B-dependent floral volatile, chiloglottone 1, for specific male wasp pollinator attraction. Chiloglottone 1 and related variants (2,5-dialkylcyclohexane-1,3-diones), represent a unique class of specialized metabolites presumed to be the product of cyclization between two fatty acid (FA) precursors. However, the genes involved in the biosynthesis of precursors, intermediates, and transcriptional regulation remains to be discovered. Chiloglottone 1 production occurs in the aggregation of calli (callus) on the labellum under continuous UV-B light. Therefore, deep sequencing, transcriptome assembly, and differential expression (DE) analysis were performed across different tissue types and UV-B treatments. Transcripts expressed in the callus and labellum (∼23,000 transcripts) were highly specialized and enriched for a diversity of known and novel metabolic pathways. DE analysis between chiloglottone-emitting callus versus the remainder of the labellum showed strong coordinated induction of entire FA biosynthesis and β-oxidation pathways including genes encoding Ketoacyl-ACP Synthase, Acyl-CoA Oxidase, and Multifunctional Protein. Phylogenetic analysis revealed potential gene duplicates with tissue-specific differential regulation including two Acyl-ACP Thioesterase B and a Ketoacyl-ACP Synthase genes. UV-B treatment induced the activation of UVR8-mediated signaling and large-scale transcriptome changes in both tissues, however, neither FA biosynthesis/β-oxidation nor other lipid metabolic pathways showed clear indications of concerted DE. Gene co-expression network analysis identified three callus-specific modules enriched with various lipid metabolism categories. These networks also highlight promising candidates involved in the cyclization of chiloglottone 1 intermediates (e.g., Bet v I and dimeric α,β barrel proteins) and orchestrating regulation of precursor pathways (e.g., AP2/ERF) given a strong co-regulation with FA biosynthesis/β-oxidation genes. Possible alternative biosynthetic routes for precursors (e.g., aldehyde dehydrogenases) were also indicated. Our comprehensive study constitutes the first step toward understanding the biosynthetic pathways involved in chiloglottone 1 production in *Chiloglottis trapeziformis* – supporting the roles of FA metabolism *in planta*, gene duplication as a potential source of new genes, and co-regulation of novel pathway genes in a tissue-specific manner. This study also provides a new and valuable resource for future discovery and comparative studies in plant specialized metabolism of other orchids and non-model plants.

## Introduction

The intriguing pollination strategy of sexual deception is now known to be used by several hundred plants species representing more than 22 genera and spanning three plant families (Bohman et al., 2016). Nonetheless, known examples are dominated by the orchids. These orchids sexually lure specific male insect pollinators by ‘semiochemicals’ that mimic the sex pheromone, with pollination often achieved during attempted copulation by the pollinator with the flower (Schiestl et al., 1999; Ayasse et al., 2003; Schiestl, 2003; Franke et al., 2009; Bohman et al., 2014; Phillips et al., 2014). Floral volatiles clearly play a pivotal role in this interaction, with the chemical diversity of the compounds involved proving to be extraordinary, despite the limited number of cases fully characterized to date (Bohman et al., 2016). For example, in European *Ophrys* orchids specific blends of the commonly occurring alkenes and alkanes are involved in the case of two bee pollinated species (Schiestl et al., 1999, 2000), whereas a specific blend in precise enantiomeric ratios of unusual keto- and hydroxy-carboxylic acids is required in the scoliid wasp pollinated *O. speculum* (Ayasse et al., 2003). Within Australian sexually deceptive orchids, where several hundred species across multiple genera are pollinated by male thynnine wasps, at least four different floral volatile chemical systems are involved in pollinator attraction: (1) Specific blends of unique cyclohexanediones, called chiloglottones, which are widely employed across *Chiloglottis* orchids (Schiestl, 2003; Franke et al., 2009; Peakall et al., 2010). (2) A specific blend of alkyl- and hydroxymethyl-pyrazines in *Drakaea glyptodon* (Bohman et al., 2014). (3) Unique (methylthio)phenols in *Caladenia crebra* (Bohman et al., 2017), and (4) A combination of two biosynthetically unrelated compounds (2-hydroxy-6-methylacetophenone and (*S*)-β-citronellol) in *Caladenia plicata* (Xu et al., 2017).

**FIGURE 1:**
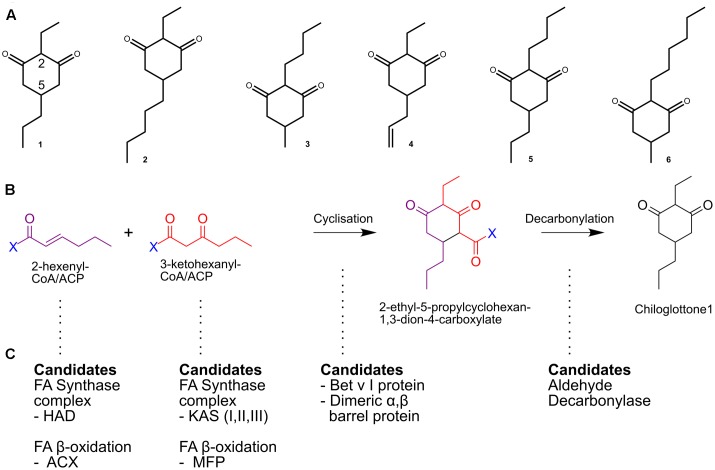
The putative chiloglottones biosynthesis pathway. **(A)** Known biologically active chiloglottones produced by *Chiloglottis* orchids. (1) 2-ethyl-5-propylcyclohexane-1,3-dione; (2) 2-ethyl-5-pentylcyclohexane-1,3-dione; (3) 2-butyl-5-methylcyclohexane-1,3-dione; (4) 5-allyl-2-ethylcyclohexane-1,3-dione; (5) 2-butyl-5-propylcyclohexane-1,3-dione; (6) 2-hexyl-5-methylcyclohexane-1,3-dione. **(B)** Presumed biosynthetic steps for chiloglottone 1 formation. **(C)** Potential metabolic routes and enzymes involved in activated precursor origins, cyclization, and decarbonylation reactions.

**FIGURE 2:**
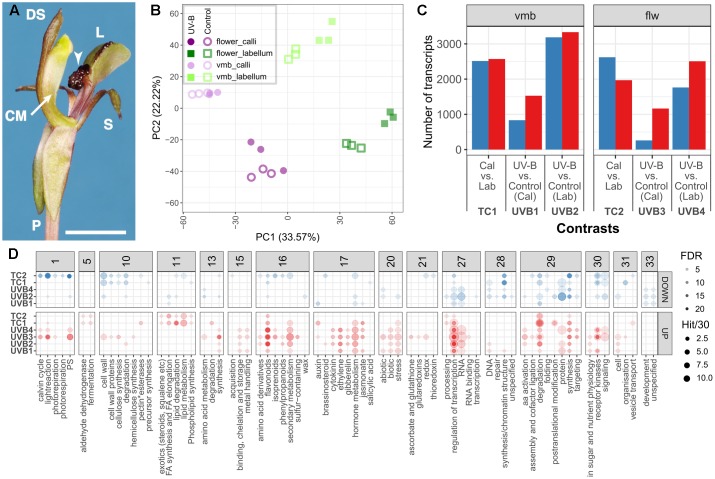
Analysis of *C. trapeziformis* expressed transcriptome. **(A)** The floral structure of *C. trapeziformis* consists of the callus (arrows), column (CM), dorsal sepal (DS), labellum (L), petals (P), and lateral sepals (S). Scale bar = 7 mm. **(B)** Principal component analysis of the transcriptome across 24 samples representing eight different tissue/treatment types. Dark purple, light purple, dark green, and light green depict callus tissues of flowers (flw), callus tissues of very mature buds (vmbs), labellum tissues of flw, and labellum tissues of vmbs, respectively. Filled circles/squares depict 2 h UV-B treated samples. Empty circles/squares depict control (covered) samples. **(C)** Summary of differentially expressed transcripts in vmb (TC1, UVB1, UVB2) and flower (TC2, UVB3, UVB4) developmental stages. The number of upregulated and downregulated in each comparison are shown. **(D)** Summary of enriched high-level (BIN depth ≤ 2) MapMan BIN categories (FDR < 0.05) describing general plant biological processes and functions for tissue and UV-B treatment comparisons in **(C)**. Red and blue colors depict enrichments for upregulated and downregulated groups of each contrast. The opacity of each circle depicts enrichments (FDR) for each enriched category. The size of the circles depicts the number of transcripts in each enriched category (See Figure for more details on opacity and size assignments). Further details can be found in **Supplementary Data 3**–**5**.

The chiloglottones (2,5-dialkylcyclohexane-1,3-diones), with six related molecules now known (labeled chiloglottone 1 to 6), represented a new class of specialized metabolites (aka secondary metabolites or natural products) when first discovered as the pollinator attractants in *Chiloglottis* orchids (Schiestl, 2003; Franke et al., 2009) (**Figure 1A**). At present, however, the genes and enzymes involved in the synthesis of this unique class of biologically active plant floral volatile constituents have not been identified. In *Chiloglottis trapeziformis*, the species in which chiloglottone 1 was first found, the third highly modified petal of the orchid, called the labellum, serves dual roles as the source of chiloglottone production (Falara et al., 2013) and as a visual and tactile mimic of the female of the pollinator (De Jager and Peakall, 2016). The densely clustered ‘insectiform’ calli structure (callus), which is attached to the labellum by a stalk (**Figure 2A**), is the sole source of chiloglottone 1 (Falara et al., 2013).

Unexpectedly, the production of chiloglottone 1 in *Chiloglottis* orchids is UV-B dependent with an optimal response at 300 nm (Falara et al., 2013; Amarasinghe et al., 2015). While UV-B light is well known to influence plant growth and development (Zhang and Björn, 2009; Heijde and Ulm, 2012), the first plant UV photoreceptor has only recently been functionally characterized. Activation of this photoreceptor, UV RESISTANCE LOCUS8 (UVR8), triggers large-scale gene expression and metabolite reprograming (Jenkins, 2009; Heijde and Ulm, 2012). Low doses of longer wavelength UV-B (>305 nm) over a short period (e.g., minutes to several hours) are known to trigger UV-B specific signaling. Meanwhile, high doses of shorter UV-B wavelength (<295 nm) over longer duration activate an additional suite of stress-responsive pathways (Jenkins, 2009). The interaction between UV-B and chiloglottone production in *Chiloglottis* is the only known example for a floral volatile compound, and the pattern of UV-B dependence is unusual (Falara et al., 2013; Amarasinghe et al., 2015). For example, *C. trapeziformis* flowers collected from the wild, containing chiloglottone 1, become depleted of chiloglottone over a period of several days in a growth cabinet lacking UV-B light. Re-exposure to natural sunlight, or artificial UV-B light, rapidly reinitiates the production of chiloglottone 1, with detection within 2 min, and measurable levels within 5 min. However, only with continuous UV-B exposure for a further 2–4 h, is the internal storage pool replenished to normal levels. While initiation of chiloglottone 1 biosynthesis requires only UV-B light, sustained chiloglottone production requires both continuous UV-B and *de novo* protein biosynthesis. Collectively, the available evidence suggests that an entirely new and unexpected UV-B dependent biochemical reaction may be involved in chiloglottone production. As a first step toward elucidating the genes and enzymes involved in the biosynthesis of chiloglottones, we report the first RNA-sequencing study of floral transcriptomes, representing different tissue and UV-B treatments, in *Chiloglottis trapeziformis*.

Chiloglottones are predicted to be built from fatty acid (FA) precursors (Franke et al., 2009; Bohman et al., 2016). For example, biosynthesis of chiloglottone 1 (2-ethyl-5- propylcyclohexan-1,3-dione) may involve the condensation of activated 3-oxohexanoic acid (C6) and 2-hexenoic acid (C6), as precursors (**Figure 1B**). In plants, such precursors are potential intermediates of both the FA biosynthesis and FA degradation (β-oxidation) pathways as ACP derivatives in plastids or as CoA derivatives in the peroxisomes, respectively (**Figure 1C**). The condensation of the activated precursors and subsequent decarbonylation may also be enzymatic involving novel plant cyclase and decarbonylase, respectively (**Figure 1C**). Thus, in this initial transcriptome study we focused on these pathways. We also search for gene duplication within the FA related pathways because gene duplication constitutes a key mechanism for the evolution of genes involved in specialized metabolism (Pichersky and Gang, 2000; Pichersky et al., 2006).

Building upon the *a priori* chemical and biochemical background to chiloglottones, here we address six key questions by in depth transcriptome analysis. Specifically we ask to what extent are changes (large- or small-scale) in biochemical and transcriptional pathways associated under (1) natural levels of UV-B and (2) in different floral tissues linked to chiloglottone 1 production? (3) Are FA biosynthesis and degradation pathway differentially regulated under (1) and (2)? (4) Are there any signatures of gene duplication in the FA biosynthesis and degradation pathway genes, and if present, are they also differentially regulated? Is there any transcriptomic evidence for (5) candidate genes involved in biochemical steps beyond precursor availability or (6) alternative biosynthetic routes to chiloglottone production?

## Materials and Methods

## Sample Collection and Plant Growth Conditions

Whole *Chiloglottis trapeziformis* plants consisting of their single very mature bud (vmb) or open flower, paired leaves and tuber, were sourced from a naturally growing colony within the Australian National Botanic Gardens (Canberra, ACT, Australia) in September 2014. Plants were potted and acclimatized in a growth chamber for a minimum of 5 days under the following conditions; 12 h day/night cycle, light intensity of 300 μmol m^-2^ s^-1^ (< 400 nm), and day/night temperatures of 20/15°C prior to UV-B experiments.

### Developmental Stage-Dependent UV-B Treatment and RNA Extraction

A low-fluence UV-B treatment was achieved using a custom made light-box following Amarasinghe et al. (2015), who also provide the criteria for defining the developmental stages we used. UV-B treatments were performed over a 2 h period on manually opened vmb and on flowers (flw) that had naturally opened in the field, but had become depleted of chiloglottone 1 in the growth cabinet over a 5–7 day period. Control samples were wrapped with aluminum foil but otherwise subjected to the same 2 h treatment. A total of 15 plants (5 plants for each biological replicate, 3 biological replicates in total) were used for each treatment. The stalked callus on the labellum, and the remaining labellum structure were immediately dissected and snap-frozen in liquid nitrogen. Extraction of total RNA was performed with the Qiagen^®^RNeasy^®^plant mini kit (Qiagen, Australia) using ∼100 mg of ground tissues. Poly(A) mRNA isolation and cDNA library construction was performed using NEBNext Poly(A) mRNA Magnetic Isolation Module and Ultra RNA Library Prep Kit for Illumina (NEB, Australia), respectively, according to the manufacturer’s instructions. Sequencing was performed at the Biomolecular Resource Facility (BRF), ANU on the Illumina HiSeq 2500 platform.

### *De Novo* Transcriptome Assembly and Differential Gene Expression Analysis

Raw FASTQ (150 bp paired-end) reads were trimmed and filtered using Trimmomatic v0.35 (Bolger et al., 2014) with the following parameters; leading, trailing, sliding window, and minlen values are 20, 20, 4:20, and 50, respectively. *De novo* transcriptome assembly was performed using Trinity v2.1.1 (Grabherr et al., 2011) with default parameters using non-redundant reads that were pooled across all sequenced libraries. Reads were multi-mapped to the assembled transcriptome using Bowtie v1.0.1 (Langmead et al., 2009) using the following parameters: -k 100, -v 3, –best, and –strata. Read summarization of clusters (hierarchically clustered contigs, referred to as transcripts) was performed using Corset v1.04 (Davidson and Oshlack, 2014) with the –g parameter. Appropriate groupings was based on our experimental design and multi-mapped reads from Bowtie as input. Independent filtering was performed to remove transcripts that were consistently expressed at low levels across the libraries (i.e., transcripts having count per million < 1.5 in > 21 experiments) prior to differential expression (DE) analysis. Analysis of DE was performed using DESeq2 v1.14 (Love et al., 2014) with default parameters in R^1^. False discovery rate (FDR) < 0.05 and absolute log2 fold change (log2FC) > 0.5 were the criteria we used to define DE transcripts between contrasts. Expression estimates of transcripts were calculated with DESeq2 v1.14 and reported as normalized or variance stabilized transformation (VST) count values (Love et al., 2014). Principal component analyses (PCAs) of the transcriptome datasets were performed in R using VST count values. Additional transcript abundance estimation, expressed as Fragments Per Kilobase of transcript per Million mapped reads (FPKM), was performed with edgeR (Robinson et al., 2009) using the longest assigned contig as the representative transcript length for normalization.

### Functional Annotation of Assembled Transcriptome

The TRAPID plant-specific pipeline (Van Bel et al., 2013) was adopted to assign functional annotations (e.g., gene ontology, GO; PLAZA gene families) to the *de novo* assembled transcriptome with the following parameters; blast_db_type = GF_REP, blast_db = gf_representatives, e_value = 10*e*-5, gf_type = HOM, func_annot = gf_besthit. TRAPID also determines potential open reading frames (ORFs) and performs frameshift correction when necessary and assigns a meta-annotation (full length, quasi full length, partial, and no annotation) for each contig. Contigs with comparable lengths to their assigned gene families receive the ‘quasi full length’ tag, and if both start and stop codons are present, a ‘full length’ is given. Otherwise, contigs are assigned as ‘partial’ or ‘no information’ when their lengths are significantly shorter lengths to their assigned gene families or no gene family is assigned, respectively. Another plant-specific pipeline, Mercator (Lohse et al., 2014), was also used to provide additional functional annotation and assignment of MapMan BIN ontology (Thimm et al., 2004). Default parameters were used with additional ‘ORYZA’ and ‘IPR’ settings enabled. Enrichment *P*-values for MapMan BIN categories in DE gene groups and gene co-expression modules were determined based on a hypergeometric distribution adjusted with FDR for multiple hypothesis correction in R, using previously established workflows (Wong et al., 2016). Emphasis were given to enriched MapMan BIN categories (FDR < 0.05) describing high-level (BIN depth ≤ 2) general plant biological processes (BPs) and functions following Klie and Nikoloski (2012). The PlantTFDB v4.0 annotation module was used for robust identification of transcription factor (TF) and TF family assignment (Jin et al., 2016).

### Gene Co-expression Network Analysis

Prior to gene co-expression network (GCN) construction, genes that do not vary significantly in at least one contrast were removed. Correlations between genes were estimated using the Spearman’s correlation coefficient (SCC) in R. The optimal cut-off was determined by inspecting the distribution of SCC based on single random sampling (*n* = 100) of 1,000 random genes as recommended by Vandepoele et al. (2009). The final GCN was visualized with Cytoscape v3.4.0 (Shannon et al., 2003). Identification of modules containing densely connected nodes within the GCN was performed using Heuristic Cluster Chiseling Algorithm (Mutwil et al., 2010) with the parameters; three-step node vicinity network and the desired cluster size ≥ 50 nodes.

### Phylogenetic Analysis

Phylogenetic analyses were performed using the MEGA7 software suite (Kumar et al., 2016) using predicted full-length protein sequences of assembled transcripts and other plant protein sequences containing the protein domain (INTERPRO) of interests from Ensembl Plants release 34^2^. Briefly, multiple sequence alignment was performed using MUSCLE, phylogenetic trees were constructed with the neighbour-joining (NJ) method with default settings in MEGA. Bootstrap analysis was performed to measure reliability of tree nodes using 1,000 replicates.

### Accession Numbers

All raw sequence reads have been deposited in NCBI Sequence Read Archive^3^ under the BioProject accession PRJNA390683 and SRA study accession SRP109328. All *de novo* transcriptome assemblies have been deposited in DDBJ/EMBL/GenBank via NCBI Transcriptome Shotgun Assembly Database^4^ under the accession GFPK00000000. The version described in this study is the first version, GFPK01000000.

## Results

### Transcriptome Sequencing and Overview of the Assembled Floral Transcriptome

RNA-seq was performed on *C. trapeziformis* to gain insights into the biochemical and regulatory pathways that are active in flowers (**Figure 2A**), and to search for clues into chiloglottone 1 biosynthesis. In total, 24 cDNA libraries representing eight different tissue/treatment types were constructed: This included the active callus versus the non-active remainder of labellum each for two developmental stages, very mature bud (vmb) and depleted flower (flw), further split into 2 h UV-B treatment and control for each tissue type and developmental stage. Across these eight types, chiloglottone 1 production only occurs in the callus of the vmb and flw type under 2 h UV-B, with chiloglottone levels higher in the flw than the vmb stage. Three biological replicates were used for each pool of the eight types of treatment. A total of 533,355,046 paired-end reads 150 bp in length, equivalent to 160 gigabases of sequence data, were obtained. Removal of adapter and low-quality sequences results in 464,624,600 high-quality PE reads, which represented 87.1% of the total sequenced reads (**Table 1**). Given the lack of *C. trapeziformis* genome, reconstruction of the floral transcriptome for the 464.6 M high-quality PE reads used a three-step strategy involving Trinity (Grabherr et al., 2011), Bowtie (Langmead et al., 2009), and Corset (Davidson and Oshlack, 2014), producing 686,243 contigs in the preliminary assembly. The final assembly consists of 221,668 contigs retained by Corset (**Supplementary Data 1**, **2**) – effectively removing many short contigs (<200 bp) while retaining the ones that have strong read support and/or shared sequence similarity (Supplementary Figures 1A,B). In addition, Corset show that 221,668 contigs can be further clustered into 146,545 clusters (transcripts). Summary statistics of the final assembly shows the average length is approximately 1,301 bp, N50 score of 1,953 bp, and a GC content of 0.4, among others (Supplementary Figure 1C).

**Table 1:** Summary of RNA sequencing analysis metrics.

Sample ID	Tissue	Developmental stage	Treatment	Raw reads^†^	Trimmed reads^†^	Total alignments^†^ (unique)	Total alignments^†^ (Multi-mapped)	NCBI Biosample ID
CT000001	Cal	flw	UV-B	12.5	9.7	9.3	42.0	SAMN07248588
CT000002	Lab	flw	UV-B	18.8	13.1	12.7	66.1	SAMN07248707
CT000003	Cal	flw	UV-B	21.9	17.9	17.3	85.4	SAMN07248685
CT000004	Lab	flw	UV-B	38.3	29.0	28.5	189.3	SAMN07248708
CT000017	Cal	flw	UV-B	16.1	12.3	11.9	60.8	SAMN07248696
CT000018	Lab	flw	UV-B	18.2	9.2	8.9	55.3	SAMN07248709
CT000005	Cal	flw	Cov	18.9	14.5	14.1	67.5	SAMN07248585
CT000006	Lab	flw	Cov	19.0	16.3	15.8	76.6	SAMN07248704
CT000007	Cal	flw	Cov	17.2	13.9	13.5	69.2	SAMN07248586
CT000008	Lab	flw	Cov	18.0	13.9	13.7	93.9	SAMN07248705
CT000019	Cal	flw	Cov	39.9	33.4	32.3	157.1	SAMN07248587
CT000020	Lab	flw	Cov	16.3	13.3	13.1	89.0	SAMN07248706
CT000025	Cal	vmb	UV-B	13.8	83.3	81.0	45.6	SAMN07248700
CT000026	Lab	vmb	UV-B	28.4	22.2	21.7	126.4	SAMN07248713
CT000027	Cal	vmb	UV-B	14.5	10.5	10.2	53.3	SAMN07248701
CT000028	Lab	vmb	UV-B	27.5	19.1	18.7	118.2	SAMN07248714
CT000029	Cal	vmb	UV-B	21.5	12.4	12.1	67.7	SAMN07248702
CT000030	Lab	vmb	UV-B	30.8	18.4	18.0	119.8	SAMN07248715
CT000031	Cal	vmb	Cov	29.1	22.9	22.3	116.2	SAMN07248697
CT000032	Lab	vmb	Cov	24.3	14.7	14.1	64.9	SAMN07248710
CT000033	Cal	vmb	Cov	27.9	21.3	20.8	108.2	SAMN07248698
CT000034	Lab	vmb	Cov	24.5	15.8	15.3	80.0	SAMN07248711
CT000035	Cal	vmb	Cov	17.7	14.5	14.1	77.2	SAMN07248699
CT000036	Lab	vmb	Cov	18.6	13.0	12.5	58.4	SAMN07248712


*vmb, very mature buds; flw, flowers; Cov, covered; Cal, callus; Lab, labellum; MM, multi-mapped; ^†^million.*

Sequence similarity searches (RapSearch2) using TRAPID (Van Bel et al., 2013) revealed that significant protein hits were found for 88,454 contigs against the PLAZA reference proteome with top matches against *Vitis vinifera* (8,339), *Oryza sativa* ssp. *indica* (7,691), *Brachypodium distachyon* (7,266), *Sorghum bicolor* (6,899), and *Glycine max* (6,878) protein sequences (Supplementary Figure 2A). In addition, 87,219 (39.3%) contigs were assigned as full-length, quasi full-length, or partial, while 134,449 (60.7%) contigs has no information assigned. Of these, 118,423 contigs contained both start and stop codons, while 19,818 and 65,811 contigs contained only stop and stop codons, respectively (Supplementary Figure 2B).

MapMan BIN categories (Lohse et al., 2014) showed higher overall representation for protein (6,574), RNA (5,277), signaling (3,216), transport (2,642), DNA (1,837), and cell (1,666) categories amongst 34,650 (15.6%) annotated contigs (Supplementary Figure 3A). The remaining 187,018 contigs are unknown and therefore classified in the ‘not assigned unknown and no ontology’ category by Mercator. As alternative annotations, gene ontology (GO) or protein domain (InterPro) were also assigned to 72,970 (32.9%) and 78,560 (35.4%) contigs, respectively. A summary of assigned GO categories as plant GO Slim categories revealed that the cellular (21.1%) and metabolic (21.2%) process within BP, binding (22.5%) and catalytic activity (17.7%) within molecular function (MF), and cell (17.2%) and intracellular (13.3%) in cellular component (CC) are amongst the top two most abundant categories (Supplementary Figure 3B). Consistent with distributions of MapMan BIN assignments, GO BP such as nucleobase-containing compound metabolic process, DNA metabolic process, protein metabolic process, transport, and CC organization were among the main terms among contigs. All structural information and functional annotations (when identified) of individual contigs were merged according to Corset’s transcripts assignments to produce the final representative annotation.

### Global Analysis of the Floral Transcriptome

The first two axes of the principal component analysis (**Figure 2B**) for the final set of 23,553 expressed transcripts (**Supplementary Data 3**) showed a clear separation of the floral transcriptome based on tissue specificity (labellum and callus, PC1) and developmental stage (vmb and flw, PC2). Similarly, UV-B treatment effects also showed clear separation in the labellum. It is further evident that there is no cross-contamination of the eight treatment types, since all formed discrete clusters including the callus which are dissected off the labellum.

Our DE analysis proceeded in the following way. First, tissue-specific differentially expressed genes between the control callus and labellum tissue of the two developmental stages: vmb (*TC1*), flw (*TC2*), were established. Next, differentially expressed genes between control and UV-B treatment in vmb and flw stages were determined. This included four contrasts: callus tissues in vmb (*UVB1*), labellum tissues in vmb (*UVB2*), callus tissues in flw (*UVB3*), and labellum tissues in flw (*UVB4*) (**Figure 2C** and **Supplementary Data 4**). A total of 12,840 unique transcripts were identified to be differentially expressed (| log2FC| > 0.5, FDR < 0.05) across the six contrasts (**Figure 2C**). To highlight biological pathways and/or genes that were biologically relevant in these comparisons, enrichment for Mapman BIN was performed using the total expressed transcriptome as background (**Figure 2D** and **Supplementary Data 5**). For example, tissues-specific differences between callus and labellum tissues (*TC1* and *TC2*) were associated with an enrichment for many lipid metabolic (BIN11), fermentative (BIN5), and protein degradation (BIN29.5) processes in significantly upregulated genes, regardless of the developmental stage. Several cell wall and DNA synthesis/chromatin structure metabolic processes were also consistently enriched in significantly downregulated genes. UV-B treatment across both developmental stage and tissue (*UVB1–4*) was characterized by an activation for pathways related to specialized metabolism (BIN16), fermentative (BIN5), hormone metabolism (BIN17), stress (BIN20), and transcriptional regulation (BIN27.3), among others.

### Natural Levels of UV-B Exposure Triggers Transcriptome Changes Associated with UVR8 Signaling

We observed large-scale transcript changes (**Figure 2C**) in the callus and labellum tissues exposed to UV-B levels that are comparable to ambient sunlight for a duration of 2 h (treatment). A portion of these transcripts were consistently upregulated (533) and downregulated (30) across the UV-B treatments (**Supplementary Data 3**). Several encode homologs of well-known UV-B response markers (Heijde and Ulm, 2012) such as EARLY LIGHT-INDUCABLE PROTEIN (ELIP1) SUPPRESSOR OF PHYA-105 1 (SPA)-related protein involved in light signaling, REPRESSOR OF UV-B PHOTOMORPHOGENESIS 2 (RUP2) involved in negative feedback of the UV-B signaling, as well as ELONGATED HYPOCOTYL 5 (HY5) and HY5 HOMOLOG (HYH), the major TF effectors of UVR8 action (Supplementary Table 1A). Concerted induction for a large number of transcripts encoding various TF families (Supplementary Figure 5) and specialized metabolism pathways centered on the shikimate, flavonoid, terpenoid, carotenoid, and green-leaf volatile pathways were also highly responsive to the UV-B treatment (Supplementary Figures 3, 4 and **Data 3**). However, neither *de novo* FA biosynthesis, β-oxidation nor other lipid metabolic pathways showed any/clear indications of concerted DE in the chiloglottone 1 producing callus tissue of vmb and flw upon UV-B treatment.

### Coordinated Regulation of Fatty Acid Biosynthesis and β-Oxidation Pathways in Chiloglottone-Emitting Callus Tissues

**FIGURE 3:**
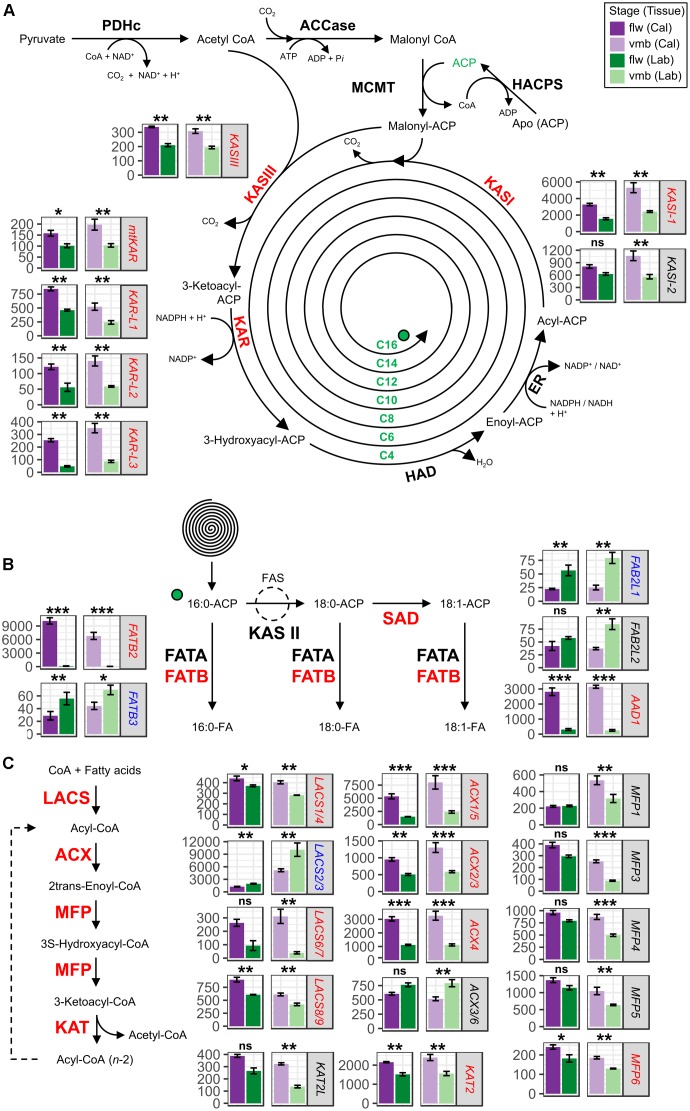
Expression of transcripts involved in the fatty acid (FA) biosynthesis and degradation pathways in labellum and callus tissues of vmb and flower developmental stages. Differentially expressed transcripts encoding enzymes involved in **(A)** FA synthesis and subsequent **(B)** elongation and desaturation in the plastids as well as **(C)** β-oxidation (degradation) in the peroxisome are depicted. Values are normalized transcript counts ± standard error. Dark purple, light purple, dark green, and light green depict callus tissue of flower stage, callus tissue of vmb stage, labellum tissue of flower stage, and labellum tissue of vmb stage, respectively. False discovery rate significance levels in each contrast are represented with symbols ^∗^(0.05 > FDR > 0.01), ^∗∗^(0.01 > FDR > 1.0*E*–10), ^∗∗∗^(FDR < 1.0*E*–10). ns represent non-statistically significant differential expression (FDR > 0.05). Biochemical reactions bolded in red depicts general modulation (upregulated or downregulated) consistency in underlying transcripts.

A large proportion of transcripts involved in plant FA biosynthesis and β-oxidation (**Figures 3A–C** and Supplementary Table 1B) were differentially expressed between callus and labellum in vmb (*TC1*) and flw (*TC2*) consistent with the observed lipid metabolism enrichment profiles (BIN11) characterized by tissues-specific differences (**Figure 2D** and **Supplementary Data 3**).

Extension of FA involves sequential condensation of two-carbon units via the FA elongase complex using acyl carrier protein (ACP) as cofactors (Li-Beisson et al., 2013). In the FA elongase complex, condensation is achieved by KAS III during the first cycle followed by KAS I in the next six cycles and finally by KAS II in the last cycle to produce 18:0 from 16:0. Transcripts encoding enzymes of the FA elongase complex involved in the condensation reactions, such as one Ketoacyl-ACP Synthase III (*CtKASIII*) and two *KASI* (*CtKASI-1* and *CtKASI-2*) were coordinately upregulated in callus tissues (**Figure 3A**). Additionally, transcripts encoding Ketoacyl-ACP Reductase (*KAR*) transcripts involved in reduction of β-ketoacyl-CoA also showed strong coordinated regulation. No DE was observed for Hydroxyacyl-ACP Dehydrase (HAD) and Enoyl-ACP Reductase (ER) transcripts. Typically, hydrolysis of long chain acyl-ACP (C16:0-C18:0) to form free FA is achieved by Acyl-ACP Thioesterase B (FATB) while hydrolysis of 18:1-ACP is performed by Acyl-ACP Thioesterase A (FATA). Majority of the 18:0-ACP pool is converted to 18:1-ACP by Stearoyl-ACP Desaturase (SAD). A large induction (>100-fold) was observed for one *FATB* transcript (*CtFATB2*) in the callus compared to the labellum (**Figure 3B**). To a lesser extent, a 10-fold increase for one *SAD* (*CtDESL1*) was observed in the callus. Nonetheless, another *FATB* (*CtFATB3*) and two *SAD* transcripts (*CtFAB2-L1/-L2*) that had lower expression were downregulated and no DE was observed for *FATA* transcripts.

The peroxisomal FA β-oxidation spiral catalyzes the complete degradation of long-chain acyl-CoA to Acetoacetyl-CoA from storage reserves and membrane lipids via sequential oxidation, hydration and dehydrogenation, and thiol cleavage (Li-Beisson et al., 2013). The first committed step involves the conversion of acyl-CoA to 2-*trans*-enoyl-CoA with Acyl-CoA Oxidase (ACX). The second involves the Multifunctional Protein (MFP) which catalyzes the formation of 3-ketoacyl-CoA from 2-*trans*-enoyl-CoA via 2-*trans*-enoyl-CoA hydratase and L-3-hydroxyacyl-CoA dehydrogenase activities. The final thiolytic cleavage of 3-ketoacyl-CoA to form acyl-CoA and acetyl-CoA is performed by Ketoacyl-CoA Thiolase (KAT). Many transcripts encoding core β-oxidation cycle enzymes were coordinately upregulated in callus compared to the labellum (**Figure 3C**). For example, three *ACX* (*ACX2/3*, *ACX1/5*, and *ACX4*) and one *KAT* (*CtKAT2*) transcript were significantly upregulated in both developmental stages. Interestingly, upregulation of five *MFP* transcripts (*CtMFP1*, *3*, *4*, *5*, and *6*) were only observed in vmb callus tissue. Downregulation of pathway transcripts were observed for one *LACS* (*CtLACS2/3*) and one *ACX* (*CtACX3/6*).

### Several Fatty Acid Biosynthesis and β-Oxidation Gene Families Have Signatures of Gene Duplication

**FIGURE 4:**
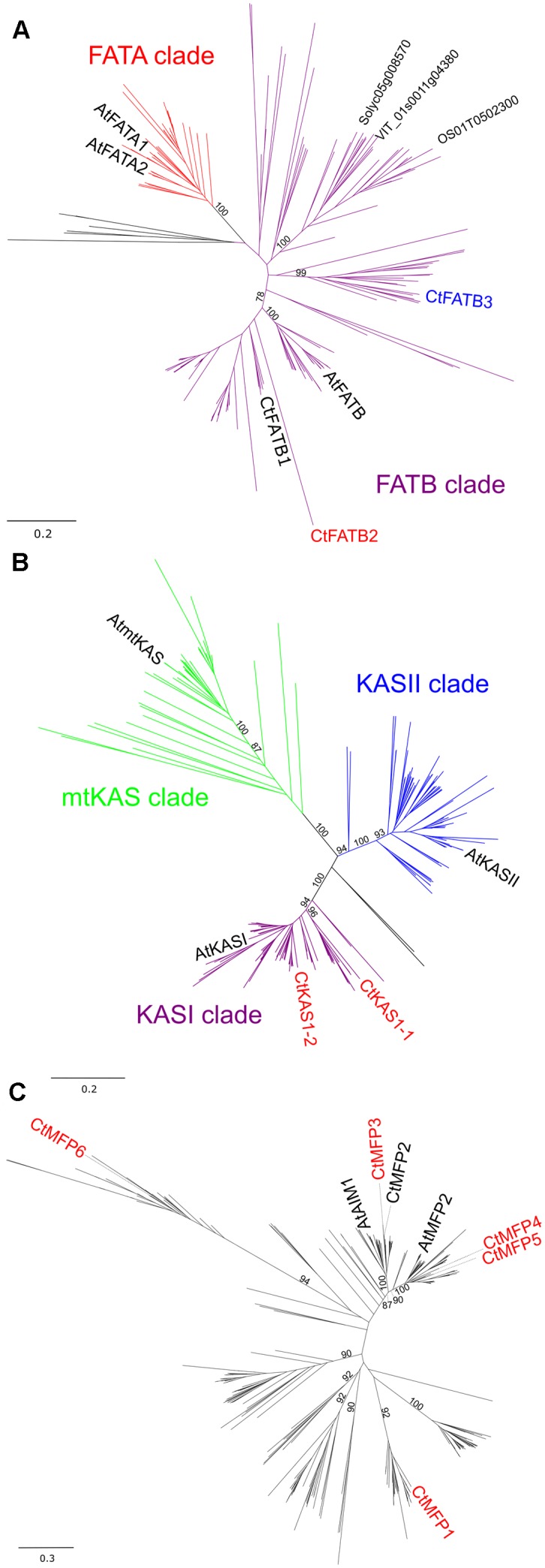
Phylogenetic tree of selected full-length protein sequences of *C. trapeziformis* with protein sequences of 41 other plants including *Arabidopsis thaliana*. Neighbor-joining trees for **(A)** Acyl-ACP Thioesterase (FAT), **(B)** Ketoacyl-ACP Synthase (KAS), and **(C)** Multifunctional Protein gene families were constructed with 226, 257, and 370 sequences, respectively. Subgroups for FAT and KAS neighbor-joining trees were defined using both bootstrap values (1,000 replicates) and by the presence functionally characterized *Arabidopsis thaliana* proteins. Bootstrap values at critical branches differentiating the subgroups/clades are only shown and others removed for overall clarity. *C. trapeziformis* sequences colored in red depicts modulation (upregulated or downregulated) consistency in TC1 and TC2 comparisons.

To determine whether gene duplication events may be present in the FA biosynthesis and degradation pathways, genome-wide comparative analysis between Arabidopsis and *C. trapeziformis* sequences was performed. Potential gene duplication were identified for FATB, KASI, and MFP gene families in *C. trapeziformis* (Supplementary Table 1B). Phylogenetic trees were also constructed for the deduced protein sequences along with other known and predicted plant FAT (A and B), KAS (I–III), and MFP sequences to ascertain the number and phylogenetic relationships among putative duplicated genes. This analysis revealed that two FATB transcripts (*CtFATB2* and *CtFATB3*) whose proteins share homology with Arabidopsis FATB represent additional paralogs to *CtFATB1* and that CpFATB3 and CpFATB2 may be divergent forms of ancestral FATB hydrolase (**Figure 4A**). In addition, one KASI duplicate/paralog (*CtKAS1-2*) sharing homology with *CtKAS1-1* as well as with Arabidopsis KASI was found (**Figure 4B**). Furthermore, of the six potential MFP transcripts identified (*CtMFP1–6*), *CtMFP2*/*3* and *CtMFP4*/*5* may represent potential gene duplicates/paralogs of *AtAIM1* and *AtMFP2*, respectively. Furthermore, *CtMFP2*, *3*, *4*, and *5* share groupings with *AtAIM1* and *AtMFP2* while *CpMFP1* and *CtMFP6* belong in two distinct groups (**Figure 4C**). Thus, there are multiple candidate gene duplicates that might participate in chiloglottone biosynthesis.

### Several Gene Co-expression Modules Are Potentially Involved in Fatty Acid/Chiloglottone Regulation

**FIGURE 5:**
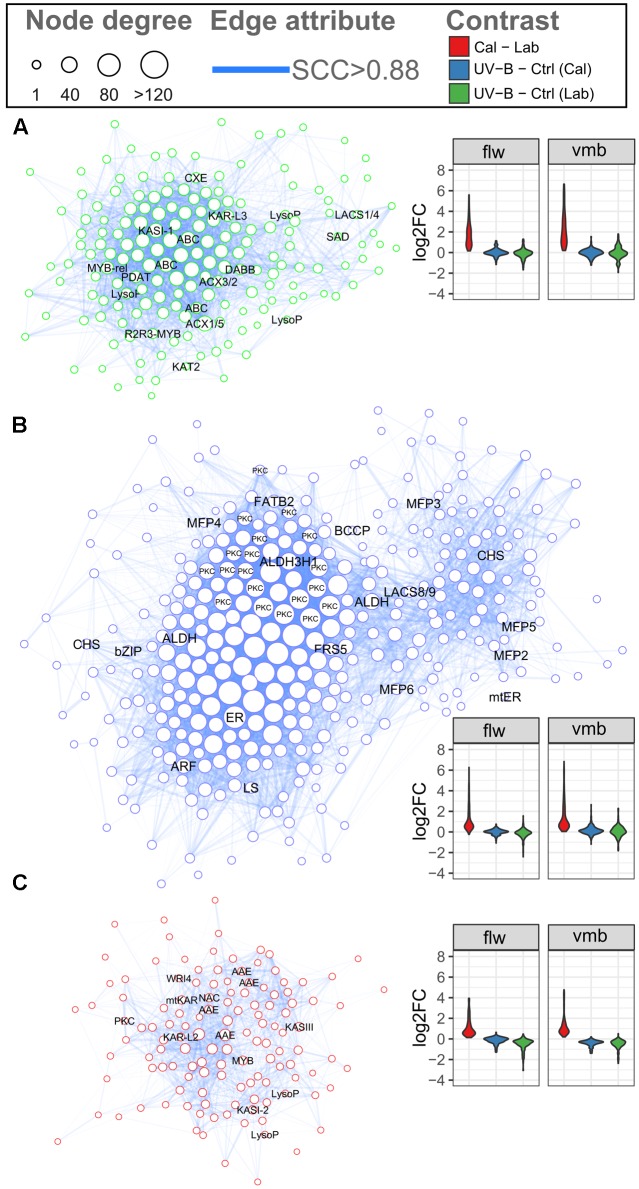
Gene co-expression network of candidate submodules involved in biosynthesis and regulation of chiloglottone 1 production. The gene co-expression network depicts genes in submodule **(A)** 7, **(B)** 13, and **(C)** 18 that share co-regulation with FA biosynthesis and β-oxidation pathway transcripts. Nodes represent genes and sizes depict the number of connections between nodes. Edges (blue) represent significant correlation (SCC > 0.88) between genes. The estimated false-positive co-expressed gene pairs in the network is 0.1%. Gene names for FA biosynthesis and β-oxidation as well as other gene candidate are displayed on the corresponding nodes. Violin plots in each submodule depict the general gene expression dynamics in vmb (TC1, UVB1, and UVB2) and flower (TC2, UVB3, and UVB4) comparisons of respective submodule. See Supplementary Figure 5 (or **Supplementary Data 3**) for details.

Genes involved in related processes often exhibit comparable expression dynamics across diverse experimental conditions (e.g., tissue/organs and stress treatments). In the case of plant specialized metabolism, GCN analysis offers a promising approach for gene function prediction in both model and non-model plant systems (Schilmiller et al., 2012; Wong and Matus, 2017). In this study, a total of 24 modules were identified (Supplementary Figure 6A and **Data 3**). Three modules with distinct expression patterns (Module, M; M7, M13, and M18) were enriched for lipid metabolism (BIN11) categories (Supplementary Figure 6B and **Data 6**). Expression of genes in these modules generally show callus-specific induction with little to no DE under UV-B treatment (Supplementary Figure 6C). To gain further insights into other biological pathways potentially involved in the regulation of chiloglottone, the FA biosynthesis and β-oxidation transcripts identified previously were used as ‘guide’ genes and all directly connected genes were extracted to form various co-expression sub-networks (submodules) (**Figures 5A–C** and **Supplementary Data 7**). These submodules all show callus-specific induction, especially submodule 7, in addition to a general downregulation of expression observed for submodule 18 for the UV-B treatment.

Submodules 7 contained seven FA biosynthesis and β-oxidation transcripts of which ACX1/5 and KASI duplicate, are genes with high connectivity (hubs) to other genes (**Figure 5A**). Additional hub genes included ABC transporters, one of which encodes an Arabidopsis peroxisomal ABC transporter homolog, involved in the uptake of fatty acyl CoA into the peroxisome. Additional genes of interests include lipases and lysophospholipases involved in triacylglycerol degradation and phospholipid signaling, respectively, and also one stress-responsive dimeric alpha-beta barrel (DABB) domain protein. FA biosynthesis genes, KAR and FATB were among the hub genes of submodule 13 (**Figure 5B**). Interestingly, there were 19 genes encoding Bet v I domain proteins in this submodule, with a large proportion acting as hubs. Two aldehyde dehydrogenase hub genes sharing homology with Arabidopsis ALDEHYDE DEHYDROGENASE 3H1 and ALDEHYDE DEHYDROGENASE 2B4 as well as two predicted polyketide synthase correlated with hub genes and several MFP genes may have important roles. In submodule 18, hub genes of interests include the FAE complex genes, KASIII, KAR, and KASI duplicate, as well as one stress-responsive DABB domain protein and several Arabidopsis acyl activating enzyme gene homologs (**Figure 5C**). In addition, the homolog of an Arabidopsis WRINKLED TF were also present in this submodule.

## Discussion

This study represents the first key step toward an understanding of the biosynthesis of the chiloglottones. We have addressed six specific questions with the overall goal to identify the transcript repertoire of *Chiloglottis trapeziformis* across the specific tissue and treatment conditions known to influence chiloglottone production and to prioritize ‘candidate’ genes for future functional studies in a hypothesis-driven manner. As no transcriptome information is currently available for *Chiloglottis trapeziformis* or any other related Australian terrestrial orchid species (>1,300 described species), reconstruction of the *C. trapeziformis* floral transcriptome was necessary. The assembled transcriptome consists of 221,668 contigs further clustered into 146,545 transcripts (Supplementary Figures 1A,B). The three-step transcriptome assembly employed in this study was previously shown to produce robust assemblies in both model and non-model organisms and improves sensitivity for detecting DE in downstream analyses (Davidson and Oshlack, 2014). Furthermore, several key assembly statistics of the complete *C. trapeziformis* floral transcriptome (Supplementary Figure 1C) such as N50 (1,953nt) and avg. length (1,301nt) were highly comparable to that of 18 other high-quality orchid transcriptomes (median N50 of 1,962nt and avg. length of 1,514nt) listed in Orchidstra database (Chao et al., 2017).

### The Callus and Labellum Transcriptome Show Highly Specialized Function

A survey against a large catalog of 25 reference plant proteomes showed that grapevines were among the top-hit species list sharing many common genes with *C. trapeziformis* albeit other monocots (rice, barley, sorghum) were also highly represented (Supplementary Figure 2A). This similarity distribution has also been reported for other orchid transcriptomes such as *Phalaenopsis* sp. (Su et al., 2011; Huang et al., 2015). In this study, we used TRAPID and Mercator, two widely adopted plant-specific annotation modules that has been specifically tuned to provide a good balance between sensitivity and specificity when annotating plant sequences with ontology categories (Bolger et al., 2017). A total of 82,564 (37.2%) of 221,668 contigs in the final assembly were successfully ascribed with GO, InterPro, and/or MapMan BIN categories (Supplementary Figures 3A,B). Many GO assignments were comparable to other floral transcriptomes of orchids (Monteiro et al., 2012; Sedeek et al., 2013). Additional MapMan BIN mappings provide a complimentary annotation scheme to GO. The remaining contigs (∼139,000) may be artifacts from *de novo* assembly (misassembly or fragments), have no coding potential, and/or lack structural similarities despite searches against the databases currently available, a phenomenon commonly reported for *de novo* assembled transcriptomes (Bolger et al., 2017). Nonetheless, these contigs may still hold other biologically relevant sequence information including novel protein domains and species-specific transcripts.

Our analysis across the 24 transcriptomes (**Figures 2B,C**) revealed that (1) the labellum and callus tissues have highly specialized transcriptomes in both buds and floral stages and (2) that the labellum transcriptomes seem to be more responsive to UV-B. The diversity of assigned categories (**Supplementary Data 3**) show that both known and potentially many novel BPs and metabolic pathways are active and functional in the flowers of *C. trapeziformis* as a whole. Enriched Mapman BIN categories (**Figure 2D**) also highlight several tissues-specific differences and UV-B responsive molecular pathways that maybe relevant to the biosynthesis and regulation of chiloglottone 1.

### UVR8 Signaling Pathway Activation and Negative Feedback Regulation

Our earlier work has lead us to hypothesize that genes encoding proteins involved in chiloglottone biosynthesis and/or maintenance may involve UVR8 signaling cascades (Falara et al., 2013). Indeed, within 5 min of low dose UV-B exposure, nuclear localization and accumulation of UVR8 can occur, leading to activation of downstream UV-B transcriptional responses (Jenkins, 2009). Strong and concerted activation of major effector genes involved in UVR8-mediated signaling such as HY5 and HYH was observed (Supplementary Table 1A and **Data 3**). Downstream metabolic pathways known to be regulated by UVR8 action in plants (Supplementary Figure 4) and known UV-B responsive TFs families such as ERF and WRKY (Supplementary Figure 5) in both callus and labellum tissues reaffirms an active UVR8-mediated UV-B transcriptional responses (Kilian et al., 2007; Jenkins, 2009). However, neither *de novo* FA biosynthesis nor β-oxidation pathway genes potentially involved in precursor supply showed indications for DE in the callus or labellum upon UV-B treatment despite active UVR8-mediated signaling (**Supplementary Data 3**).

The absence of differential UV-B regulation in the FA pathways previously predicted to directly (or indirectly) provide the substrates for chiloglottone production (Franke et al., 2009; Bohman et al., 2016) is of interest. We have uncovered a similar situation in the biosynthesis of floral volatiles in the case of *Caladenia plicata*. Here, the biosynthesis of one key active semiochemical, the monterpene (*S*)-β-citronellol, proceeds via a novel multistep route in which only two of the three genes involved its biosynthesis are differentially expressed between the sepal tips (active) and column (non-active) tissues (Xu et al., 2017). Our findings in this present study, suggest that not all steps involved in chiloglottone 1 biosynthesis are UV-B responsive and that precursor supply (i.e., activated 3-oxohexanoic acid and 2-hexenoic acid) is not limiting in the callus. Indeed, this result is consistent with the known rapid (within 2 min) production of detectable chiloglottone 1 following UV-B exposure (Amarasinghe et al., 2015). Nonetheless, regulation of chiloglottone 1 production might still require UV-B for the cyclization of activated precursors via enzymatic or non-enzymatic means (Falara et al., 2013; Amarasinghe et al., 2015).

Despite the absence of evidence for strong UV-B regulation of the candidate chiloglottone pathways, we cannot discount the involvement of negative feedback regulation on transcriptional and biosynthetic pathways during UVR8-mediated signaling. Activation of *RUP2* gene expression has been recorded as early as 1 h upon UV-B treatment in Arabidopsis (Gruber et al., 2010) and at higher UV-B irradiances to prevent hyper-activation of UVR8 signaling (Heijde and Ulm, 2012). Strong and consistent induction of *RUP2* was observed (**Supplementary Data 3**) suggesting that negative feedback regulation of UVR8-mediated signaling may have occurred before the end of the 2 h UV-B treatment and therefore the transcripts involved in chiloglottone production may have already become reduced. Biochemical evidence for negative feedback control of chiloglottone 1 production is well established, with rapid initial production in minutes following UV-B exposure in depleted flowers being followed by a production plateau with prolonged exposure of >3 h and at higher UV-B irradiances (Amarasinghe et al., 2015). Further studies assessing the expression of putative pathway genes in shorter time intervals of UV-B exposure (e.g., 5 min intervals) will be needed.

### Tissue-Specific Expression of Fatty Acid Genes (and Paralogs)

Several key FA biosynthesis and β-oxidation steps involved in the production of activated precursors were highly induced in the callus compared to the labellum. More importantly, coordinate regulation of entire pathways were observed (**Figures 3A,B**). This observation is consistent with the current hypothesis of chiloglottone 1 biosynthesis *in planta*, where FA intermediates have a likely role in its formation (Franke et al., 2009; Bohman et al., 2016) and is consistent with the callus-specific production of chiloglottone 1 in *C. trapeziformis* (Falara et al., 2013; Amarasinghe et al., 2015).

The presence of gene duplication events for FA related pathways (**Figures 4A–C** and Supplementary Table 1B) may also have implications for chiloglottone biosynthesis. Evidence for neo- and/or sub-functionalization events driving adaptive changes in alkene (semiochemical) production involved in pollinator attraction and reproductive isolation have been presented for SAD homologs (SAD1–6) in sexually deceptive *Ophrys* orchids (Schlüter et al., 2011; Xu et al., 2012; Sedeek et al., 2016). For example, expression of two SAD homologs (*SAD1*/*2*) were associated with differences in 9- and 12-alkene production between *O. garganica* and *O. sphegodes* (Schlüter et al., 2011; Xu et al., 2012) while changes to the subcellular localization and enzyme substrate specificity of SAD5 determines metabolic fates of precursors to 7-alkenes in *O. exaltata* (Sedeek et al., 2016).

### Enzymes Potentially Capable of Producing Activated 3-Oxohexanoic Acid Precursor

Transcripts encoding enzymes that may directly participate in the production of activated 3-oxohexanoic acid, one of the two postulated chiloglottone 1 precursors (**Figure 1B**), include KASI from FA biosynthesis and MFP in the FA degradation pathway (Li-Beisson et al., 2013). Indeed, two *KASI* (*CtKAS1-1*/*-2*) and five *MFP* transcripts (*CtMFP1*/*3*/*4*/*5*/*6*) were upregulated in the callus compared to the labellum (**Figures 3A,C**). This indicates potential for an over-production of activated β-ketoacyl (3-ketoacyl) units which may include 3-oxohexanoic acid-CoA/-ACP precursors.

Relative to Arabidopsis considerable expansion of MFP gene families were observed for *C. trapeziformis* (**Figure 4C**). In Arabidopsis, two MFP isoforms exist, namely AtAIM1 and AtMFP2 (Rylott et al., 2006; Arent et al., 2010). Both AtAIM1 and AtMFP2 have broad chain length-specific dehydrogenase activities but differ in substrate preference of their corresponding hydratase activities, whereby AtAIM1 have shorter substrate preference (e.g., C4:0, C6:0) and vice versa (e.g., C12:0) for AtMFP2 (Arent et al., 2010). Neo- and/or sub-functionalization of MFP paralogs might evolve novel hydratase and dehydrogenase activities, favoring short-to-medium chain length (i.e., C6:0, C8:0) enoyl-CoA and hydroxyacyl-CoA substrates suited for chiloglottone 1 biosynthesis. Similarly, the additional copy of KASI (**Figure 4B**) might favor shorter chain length (e.g., C4:0) activated Acyl-ACP, given that KASI is important for the subsequent reactions including condensation of 3-ketobutyrl (C4:0)-ACP to 3-Ketohexanoyl (C6:0)-ACP.

Evidence for a novel class of KAS, capable of catalyzing the formation of 2,5-dialkylcyclohexane-1,3-diones compounds using the same mechanism proposed for chiloglottone formation (Franke et al., 2009), have recently been reported in bacteria (Fuchs et al., 2013; Mori et al., 2016). For example, the enzyme DarB catalyzes the formation of 4-carboxy-2,5-dialkylcyclohexane-1,3-diones (Fuchs et al., 2013), via the condensation of activated β-palmitoyl with 2-butenoyl while the enzyme StlD catalyzes the formation of isopropyl styryl-2,5-dialkylcyclohexane-1,3-diones (Mori et al., 2016) using activated isovaleryl with 5-phenyl-2,4-pentadienoyl. Although the CtKASI paralog and bacteria DarB and StlD share low sequence similarities, further studies will be needed to ascertain whether *C. trapeziformis* KASI paralog have evolved additional capabilities independently to perform these condensation reactions.

### Enzymes Potentially Capable of Producing Activated 2-Hexenoic Acid Precursor

Supply of activated 2-hexenoic acid, a second postulated precursor for chiloglottone 1, may include HAD and ACX activities from the FA biosynthesis and degradation pathways, respectively (**Figure 1B**). Three ACX transcripts were upregulated in the callus compared to the labellum potentially implicating ACX activities for activated precursor supply compared to HAD, where no DE were observed (**Figure 3C**). In Arabidopsis, six ACX isoforms (ACX1–6) have been identified, many of which have varying chain-length substrate specificities but are often overlapping (Graham, 2008). Although comparative sequence homologies with Arabidopsis resolved the identity for one ACX transcript (CtACX4), the other two ACX transcripts remains unresolved, and may be homologs of AtACX2/3 and AtACX1/5, respectively (**Supplementary Data 3**). Nonetheless, Arabidopsis ACX4 possess short-to-medium (C4:0-C8:0) acyl-CoA oxidase activities (Hayashi et al., 1999), and the same may be implied for CtACX4 in supplying the 2-hexenoic acid precursor. Upregulation of CtACX1/5 and CtACX2/3 transcripts (**Figure 3C**) may relate to a higher medium-to-long acyl-CoA oxidase (ACX1–3) activities (Hooks et al., 1999; Froman et al., 2000) in the callus, thus ensuring continuous passage through the β-oxidation spiral prior to CtACX4 activities.

### Enzymes Potentially Involved in Mid-Cycle Termination of Iterative FA Biosynthesis and/or Degradation Pathway

A mid-cycle termination of the otherwise iterative FA biosynthesis to C16/C18 and/or degradation pathways to C2 has been hypothesized as a possible way to divert activated FA precursors at the appropriate carbon length of C6 for chiloglottone 1 production (Bohman et al., 2016). Although, just how this might be achieved is unknown. In this context, the coordinated regulation of several FATB genes observed across our transcriptome is of interest (**Figure 3B**). Competition between acyl chain elongation and premature cleavage of acyl-ACP are known for several plant species (Voelker and Kinney, 2001) especially the ones that accumulate shorter chain FA (e.g., C8–C14) in specific tissues such as oil palm and coconut (Jing et al., 2011; Dussert et al., 2013). These plants often contain duplicated FATB genes that have neofunctionalized to hydrolyze shorter (C8:0-C14:0) acyl-ACP substrates. For example, premature cleavage of acyl-ACP by coconut (CnFATB1 and CnFATB3) and oil palm (EgFATB3) FATB paralogs, are involved in the accumulation of medium and short chain FA in their seeds (Jing et al., 2011; Dussert et al., 2013). In addition, tissue-specific differential regulation of FATB genes in oil palm also determines the type of FA accumulated between fruit tissues (Dussert et al., 2013). Endosperm-specific *EgFATB3* expression determines accumulation of shorter chain FA in the endosperm and mesocarp-specific *EgFATB2* determines long chain FA in the mesocarp.

Two potential FATB paralogs, *CtFATB2* and *CtFATB3* were identified (**Figure 4A**) in *C. trapeziformis*, with *CtFATB2* highly expressed in the callus compared to the labellum (**Figure 3B**). It is thus possible, that a combination of tissue-specific regulation and potential shorter chain length specificities for *CtFATB2* may favor early acyl chain elongation termination similar to that observed for oil palm (Dussert et al., 2013). Thus, preventing further chain elongation may constitute another route for chiloglottone 1 precursor accumulation.

### Fatty Acid Co-regulated Genes on Chiloglottone 1 Production

In this study, GCN analysis identified three modules that were consistently induced in the callus compared to the labellum (**Figures 5A–C**). These modules were enriched for various lipid metabolism categories and contained several FA biosynthesis and β-oxidation pathway transcripts (Supplementary Figure 6B and **Data 6**). GCNs showing tissue-specific coordinated regulation of numerous (not just some) FA pathway transcripts and other lipid metabolism pathways are not entirely uncommon. Such are known elsewhere where unique FA’s are major target metabolites in different oil palm fruit tissues (Guerin et al., 2016). Using FA biosynthesis and β-oxidation transcripts as ‘guide’ genes, many genes encoding Bet v I domain proteins, aldehyde dehydrogenases, polyketide synthase, and TFs were not only strong co-regulated with the them but showed high connectivity to other genes within the submodules (**Figures 5A–C**). Some of these genes are of interest for their involvement in the final step(s) in chiloglottone 1 biosynthesis involving cyclization and decarbonylation reactions (**Figure 1B**).

### Co-regulated Enzymes Potentially Involved in Cyclization of Chiloglottone 1 Precursor

Plant Bet v I proteins belongs to a large superfamily of structurally related proteins, several of which have been reported to have cyclization activities and broad-spectrum ligand binding capabilities (Radauer et al., 2008). Many Bet v I domain proteins were not only correlated with FA pathways genes (e.g., FATB paralog) but with many other genes in submodule 13 (**Figure 5B**). The latter suggest that Bet v I domain proteins could be involved in chiloglottone 1 production by binding to short-to-medium chain length FA during storage or directly participating in the condensation reaction of activated precursors itself. DABB proteins are also candidate cyclases in chiloglottone 1 prioritized in submodules 7 and 18 (**Figures 5A,C**). To date, only one plant DABB gene has been characterized. The *Cannabis sativa* olivetolic acid cyclase was demonstrated to catalyze the cyclization of pentyl diacetic acid lactone intermediate to olivetolic acid, a key precursor in the cannabinoid biosynthetic pathway (Gagne et al., 2012; Yang et al., 2016). Co-regulation of one DABB gene with KASI-1 duplicate in submodule 7 (**Figure 5A**) suggest that these two proteins are prime cyclization candidates of activated FA precursors.

### Alternative Pathways for Activated Precursor Supply

Roles for co-regulated ALDEHYDE DEHYDROGENASE 3H1 may provide an alternative means for activated 2-hexenoic acid supply. Arabidopsis ALDEHYDE DEHYDROGENASE 3H1 is capable of oxidizing a wide range of aldehydes into corresponding carboxylic acids including *trans*-2-hexenal (Stiti et al., 2011). Co-regulated AAE transcripts from other tissue-specific submodules may assist in the activation of 2-hexenoate (**Figure 5C**). Additionally, co-regulation of *ALDEHYDE DEHYDROGENASE 2B4* gene suggest the involvement of the pyruvate dehydrogenase (PDH) bypass pathway for acetyl-CoA supply (Wei et al., 2009) given the absence of PDH gene differentially expression and the need for acetyl-CoA substrates for chain elongation. Therefore, the aerobic fermentation pathway may be an important alternative source for the acetyl-CoA precursors needed for FA biosynthesis in the callus tissues, similar to the alternative substrate sources reported in oil palm mesocarp tissues (Guerin et al., 2016).

### Transcription Factors Potentially Involved in the Regulation Chiloglottone 1 Production

Transcriptional networks controlling phenylpropanoid/benzenoid and terpenoids production in flowers involving coordinated regulation of entire biosynthetic networks are well known (Muhlemann et al., 2014). Likewise, TFs that show tissue-specific co-regulation with ‘guide’ genes have been identified in this study. The homolog of Arabidopsis WRINKLED 4 homolog known to coordinate tissue-specific regulation of FA biosynthesis pathways genes in Arabidopsis (To et al., 2012) was among the TFs highlighted in our network (**Figure 5C**). Co-regulation of FA biosynthetic genes such as KASIII, KASI, and KAR with WRINKLED 4 homolog coincides with known targets of Arabidopsis WRINKLED 4 (To et al., 2012) also suggest an importance of TFs in coordinating key FA biosynthesis and β-oxidation steps in a tissue-specific manner.

## Conclusion

In this study, we show that coordinated induction of entire FA biosynthesis and β-oxidation pathways occurs in chiloglottone-emitting callus. This finding supports the current hypothesis of chiloglottone 1 biosynthesis *in planta* via FA intermediates as precursors and matches the tissue-specific distribution of chiloglottone 1. Potential gene duplication events for FATB, KASI, and MFP gene families were also identified with all three gene family paralogs showing tissue-specific differential regulation. However, neither *de novo* FA biosynthesis, β-oxidation nor other lipid metabolic pathways showed any indications of coordinated DE upon UV-B treatment despite strong evidence for UVR8-mediated signaling and downstream transcriptome changes irrespective of tissue types. Nonetheless, we cannot yet rule out the involvement of negative feedback regulation via UVR8, in the negative feedback control of chiloglottone 1 production that has been observed in the flower. GCN analysis identified three callus-specific modules enriched with various lipid metabolism categories. These network highlight potential candidates involved in the biosynthesis and transcriptional regulation of chiloglottone for future functional analyses. Future integration of metabolome data with the existing transcriptome datasets also promises to not only advance our understanding of the complexity of metabolic control and feedback loops in chiloglottone 1 production, but is also expected to yield new insights into the largely unexplored metabolism underpinning the diverse semiochemicals involved in sexual deception. Beyond the primary goal, this study provides a new and valuable resource for comparative studies in plant specialized metabolism of other orchids and non-model plants.

## Author Contributions

DW participated in the design of the study, analyzed the data, and wrote the paper. RA and RE performed the experiments. CR-D assisted in the data analysis. RP and EP secured funding, conceived and designed the study, coordinated the experiments and data analysis, and co-wrote the paper. All authors have read and approved the paper.

## Conflict of Interest Statement

The authors declare that the research was conducted in the absence of any commercial or financial relationships that could be construed as a potential conflict of interest.

## Footnotes

1 http://www.r-project.org

2 http://plants.ensembl.org/index.html

3 http://www.ncbi.nlm.nih.gov/sra

4 https://www.ncbi.nlm.nih.gov/genbank/tsa/

## Supplementary Material

**DATA 1 |** Transcripts and all representative contigs – clustered by Corset (Davidson and Oshlack, 2014) – with associated open reading frame sequence predicted using TRAPID (Van Bel et al., 2013) in the final *C. trapeziformis* floral transcriptome assembly. Each transcript is represented as Cluster-ID and may have more than one contig associated with them.

**DATA 2 |** Transcripts and all representative contigs – clustered by Corset (Davidson and Oshlack, 2014) – with associated translated protein sequences based on the open reading frame sequences predicted using TRAPID (Van Bel et al., 2013) in the final *C. trapeziformis* floral transcriptome assembly. Each transcript is represented as Cluster-ID and may have more than one contig associated with them.

**DATA 3 |** Comprehensive annotation for all expressed transcripts in the final *C. trapeziformis* floral transcriptome assembly. Columns A – AS lists the transcript abundance (expressed as normalized transcript counts) in all 24 sequenced libraries (Columns B–Y), differential expression comparisons statistics for treatments UVB1–4 and TC1–2 (Columns Z–AK), annotation information gathered from TRAPID, Mercator MapMan BIN assignments (Lohse et al., 2014), UniProt homology searches, plantTFdb annotation (Jin et al., 2016), and Arabidopsis orthologs (Columns AL–AR), module assignments by HCCA (Mutwil et al., 2010) (Column AS), for each transcript. For alternative transcript abundance estimation – variance stabilized transformed or log2-transformed Fragments Per Kilobase of transcript per Million mapped reads – see **Supplementary Data 8**.

**DATA 4 |** Differential expression statistics generated by DESeq2 (Love et al., 2014) for treatments UVB1–4 and TC1–2. Only differentially expressed transcripts (| log2FC| > 0.5, FDR < 0.05) are shown. Columns B–H depict the baseMean, log2FoldChange, lfcMLE, lfcSE, stat, pvalue, padj (FDR) for each transcript between comparisons.

**DATA 5 |** Detailed enrichment results for MapMan BIN categories for upregulated (U) and downregulated (D) groups in comparisons TC1–2 and UVB1–4. The total number of transcripts with a BIN assignment in each comparison groups (x), total transcripts in background with the assigned BIN (X), total transcripts in each comparison groups (n), and the total expressed transcripts as background (N), raw enrichment *P*-value and adjusted *P*-value (false discovery rate, FDR), BIN ID and description, and transcripts assigned to the enriched (FDR < 0.05) BIN category are shown.

**DATA 6 |** Detailed enrichment results for MapMan BIN categories for transcripts in HCCA modules. See **Supplementary Data 5** caption for further details.

**DATA 7 |** Description of submodules 7, 13, and 18 gene co-expression network. Nodes A and B represent genes and the Spearman Correlation Coefficient value represent the correlation relationships between them. The node degree for each genes are also indicated to highlight potential ‘hub’ genes with high connectivity with other genes. See **Supplementary Data 3** for comprehensive gene annotation.

**DATA 8 |** Variance stabilized transformed (Love et al., 2014) and log2-transformed Fragments Per Kilobase of transcript per Million mapped reads (Robinson et al., 2009) values in all 24 sequenced libraries for all expressed transcripts in the final *C. trapeziformis* floral transcriptome assembly.
