# Supplementary material for: Tissue-Specific Floral Transcriptome Analysis of the Sexually Deceptive Orchid Chiloglottis trapeziformis Provides Insights into the Biosynthesis and Regulation of Its Unique UV-B Dependent Floral Volatile, Chiloglottone 1
Source: Front Plant Sci. 2017 Jul 19;8:1260. doi: 10.3389/fpls.2017.01260 (PMC5515871; doi:10.3389/fpls.2017.01260)

Supplementary Figures

Supplementary Figure 1. Distribution of contig frequency relative to length (nt) in the transcriptome assemblies. The bar graph show the number of contigs that present in the (A) initial assembly and (B) the final assembly (inset) after Corset contig clustering. In **A**, the total number of contigs removed by Corset in each bin length is indicated. The bin width in **A** and **B** is 200nt. (C) Summary key metrics of the final *C. trapeziformis* floral transcriptome assembly encompassing 146,545 transcripts that were hieratically clustered by Corset.

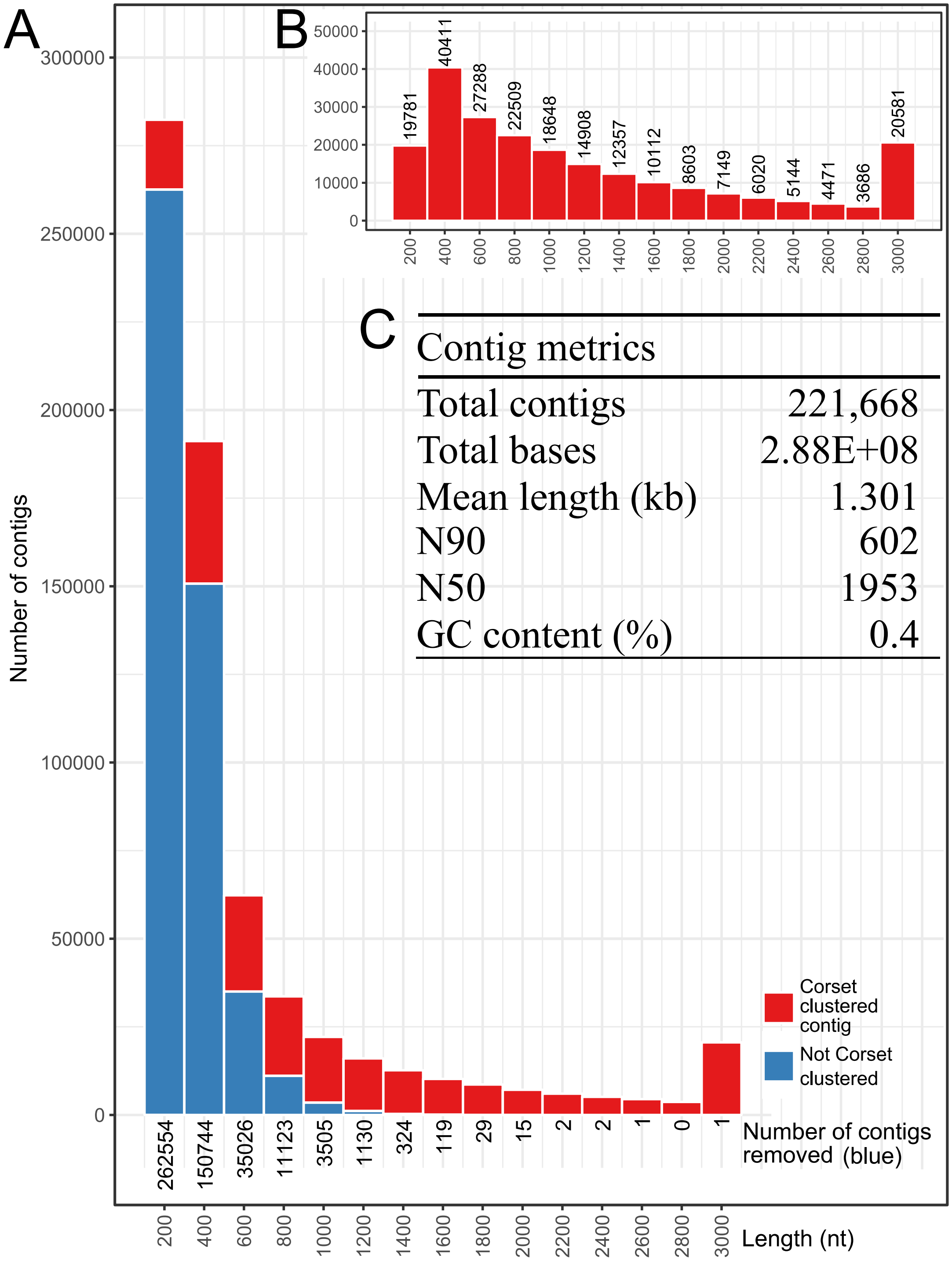

Supplementary Figure 2. Sequence similarity and coding potential distribution of the final transcriptome assembly. (A) Distribution of contigs with significant protein hits against the PLAZA reference proteome of 25 species with a sequenced genome (http://bioinformatics.psb.ugent.be/plaza/). (B) Meta-annotation assignments determined by TRAPID (e.g. Full length, No information, Partial, and Quasi Full Length) for each contig in the final transcriptome assembly. N:N, N:Y, Y:N, and Y:Y represent contigs that have no stop and start, no start but contain a stop, contain a start but have no stop, and the ones that have both start and stop codons, respectively. See **Supplementary Data 3** for full details.

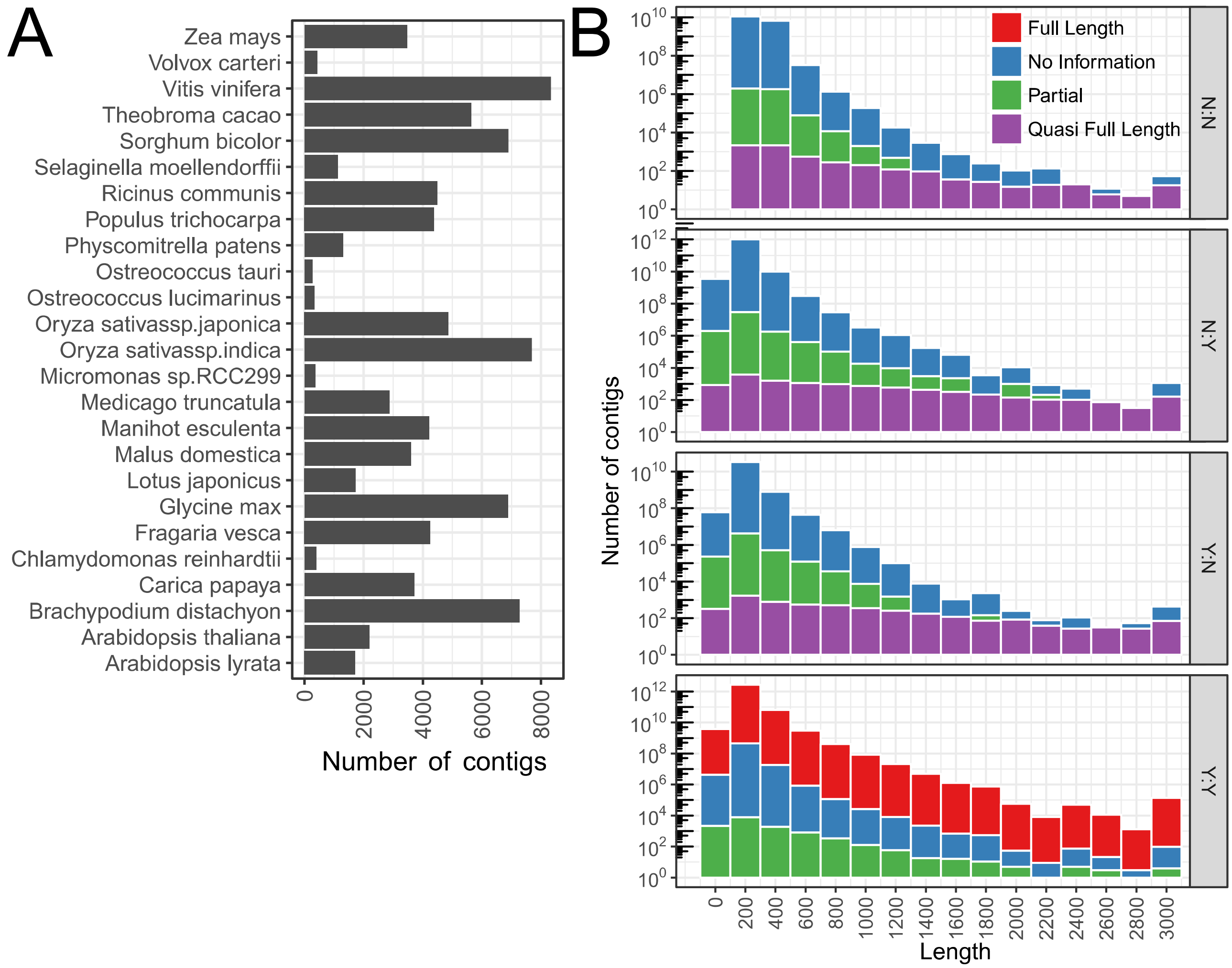

Supplementary Figure 3. Functional categories of the final transcriptome assembly. Distribution of contigs across (A) MAPMAN BIN and (B) gene ontology SLIM functional categories covering Biological Processes (BP), Cellular Component (CC) and Molecular Function (MF). For **A** and **B**, remaining contigs that are unknown, and therefore not assigned to any categories, are not plotted.

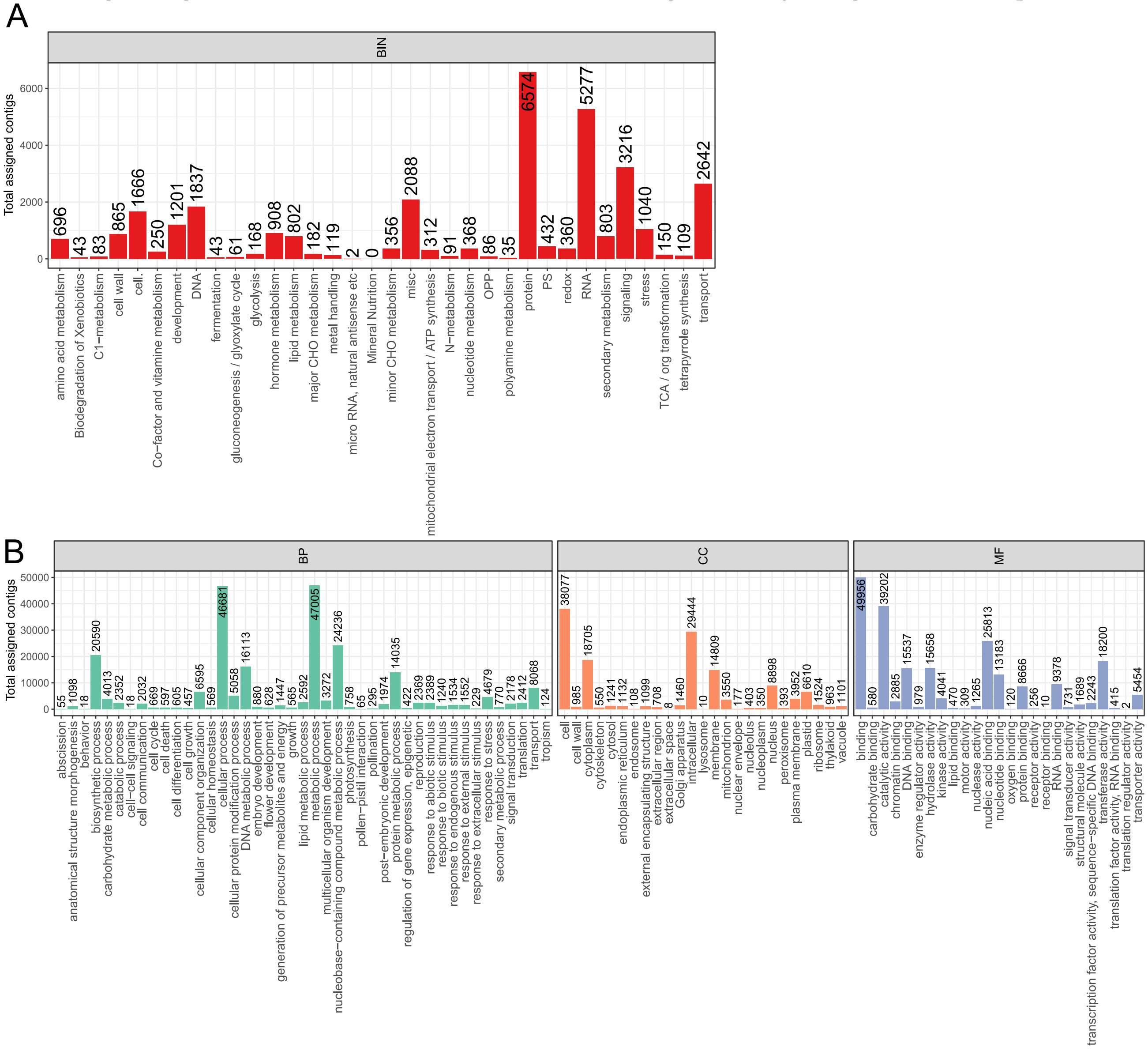

Supplementary Figure 4. Differential expression of specialized metabolism pathways under UV-B centered on the shikimate, flavonoid, terpenoid, carotenoid, and green-leaf volatile pathways. Highly responsive UV-B transcripts encoding structural enzymes for phenylalanine, phenylpropanoid, and flavonoid biosynthesis includes Arogenate dehydratase (CtADT), Phenylalanine ammonia-lyase (CtPALa and CtPALb), Flavonoid 3′-hydroxylase (CtF3′Ha, CtF3′Hb). Isoprenoid and derivative pathways genes including one terpene synthase transcript implicated in monoterpene formation, two violaxanthin de-epoxidase (CtVDEa and CtVDEb) involved in the violaxanthin cycle, and two 9-cis-epoxycarotenoid dioxygenase (CtNCEDa and CtNCEDb) catalyzing the rate-limiting step of ABA biosynthesis, were also highly responsive to UV-B. The lipoxygenase and jasmonic acid biosynthesis pathway, with one lipoxygenase (Ct13-LOXb), one allene oxide cyclase, and one 12-oxophytodienoic acid reductase transcript also responded to UV-B. (**Supplementary table 1A**). Red and blue color shades indicate higher positive and lower negative log2FC differential expression value of each transcript, respectively at each UV-B contrast. X signifies no differential expression. Bold protein labels in red represents corresponding biosynthetic steps that are consistently upregulated in UV-B treatment compared to controls (covered). See **Supplementary Data 3** for more information on transcript abundance, differential expression, and full annotation.

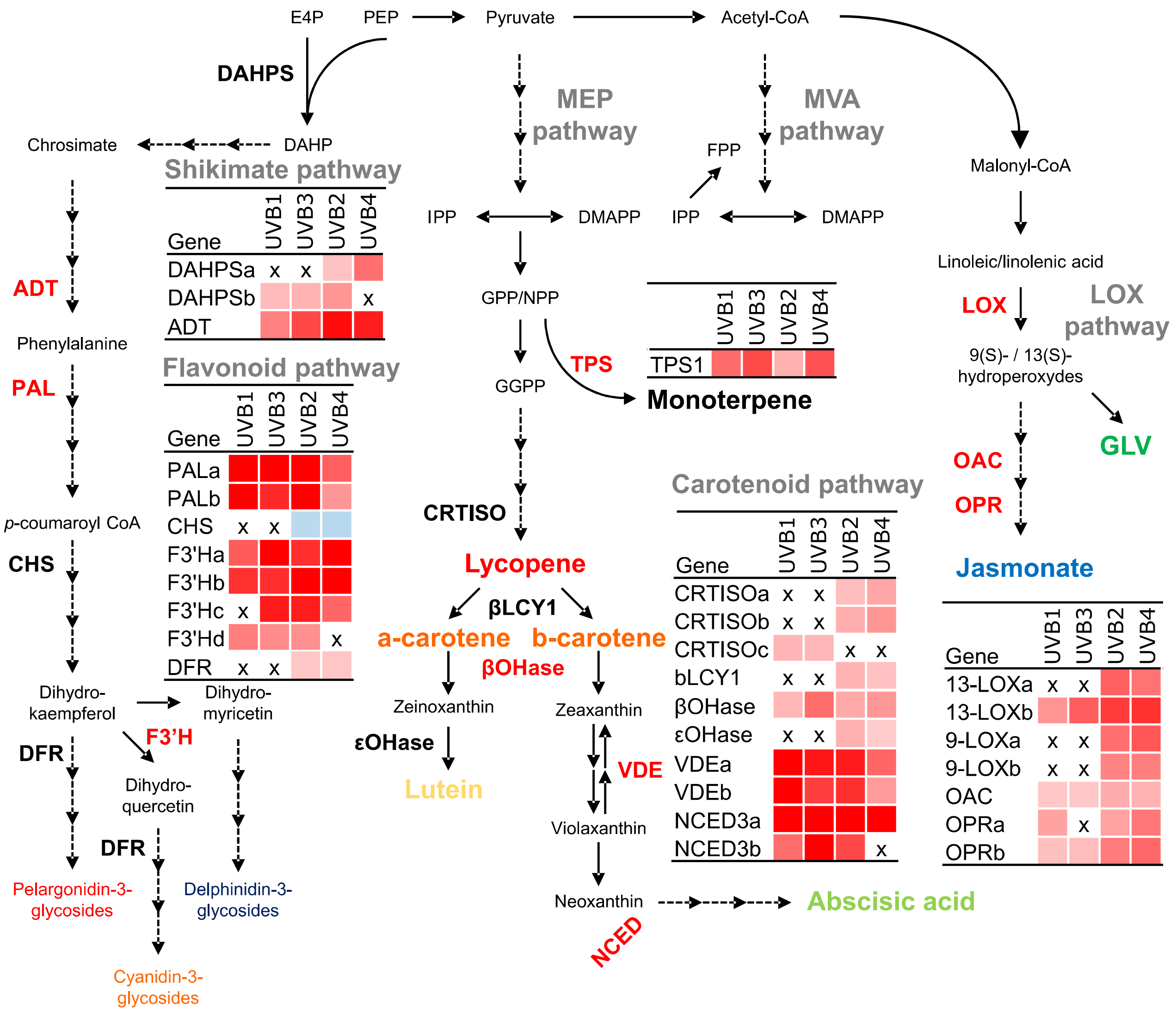

Supplementary Figure 5. Responses of differentially expressed transcription factors, grouped according to their respective families, in at least one UV-B treatment (UVB 1 – 4). Concerted induction for a large number of transcripts encoding transcription factor that are highly responsive to UV-B involves many WRKY (15 transcripts), AP2/ERF (14 transcripts), bHLH (7 transcripts), and MYB/MYB-related (7 transcripts) TF families. See **Supplementary Data 3** for more information on transcript abundance, differential expression, and full annotation.

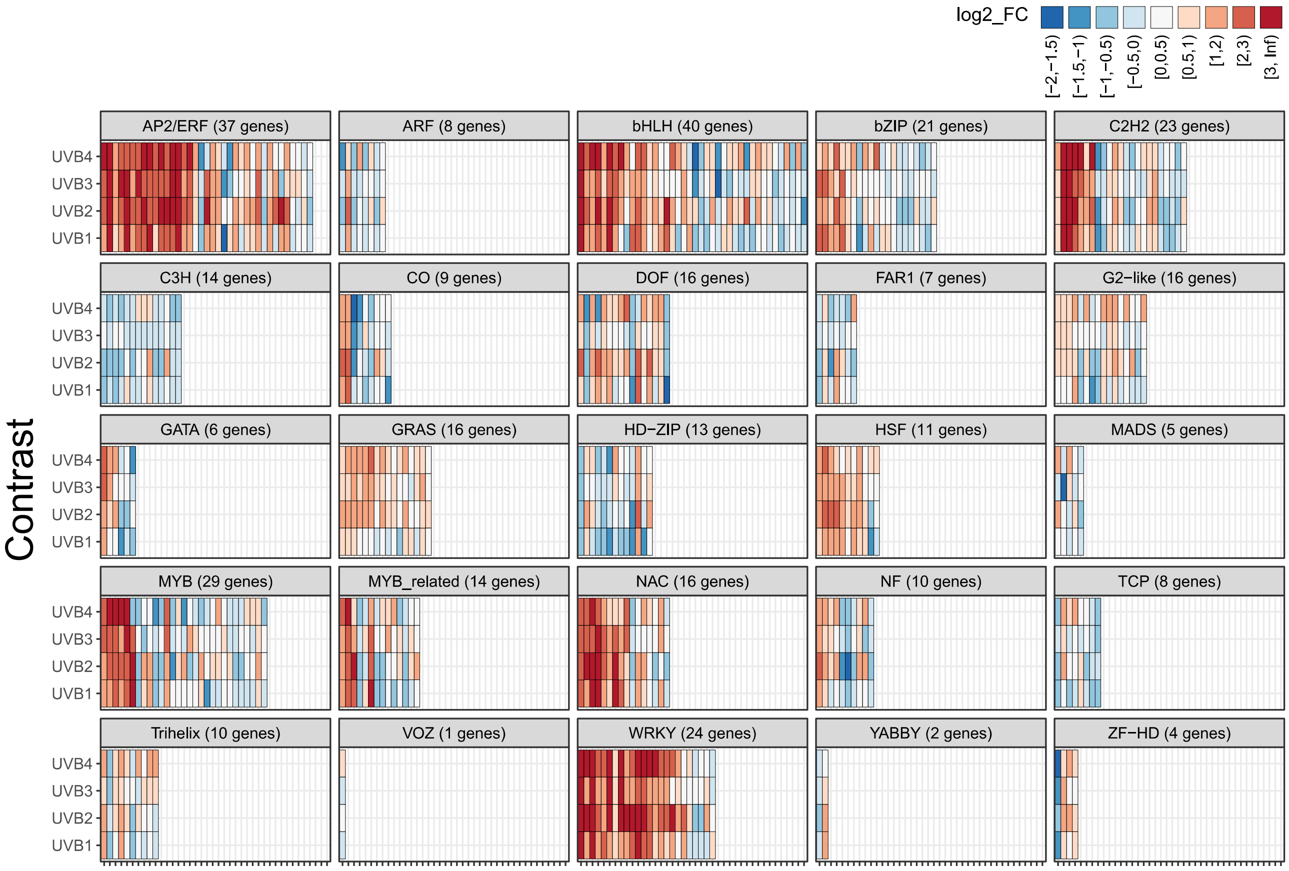

Supplementary Figure 6. Gene co-expression network encompassing 9,821 nodes (genes) and 136,319 edges (relationships) using a SCC threshold of 0.88. The estimated false-positive co-expressed gene pairs in the network is 0.1%. Twenty-four modules with distinct expression patterns were identified with Heuristic Cluster Chiseling Algorithm (Mutwil et al. 2010). (A) Modules 7, 13, and 18 enriched with lipid metabolism pathways are indicated. (B) Detailed enrichment results for Mapman BIN categories for all 24 modules using the total expressed transcriptome as background. See **Supplementary Data 6** for more information of full enrichments results. (C) Gene expression dynamics of all 24 modules based on their differential expression patterns in very mature bud (TC1, UVB1, and UVB2) and flower (TC2, UVB3, and UVB4) comparisons.

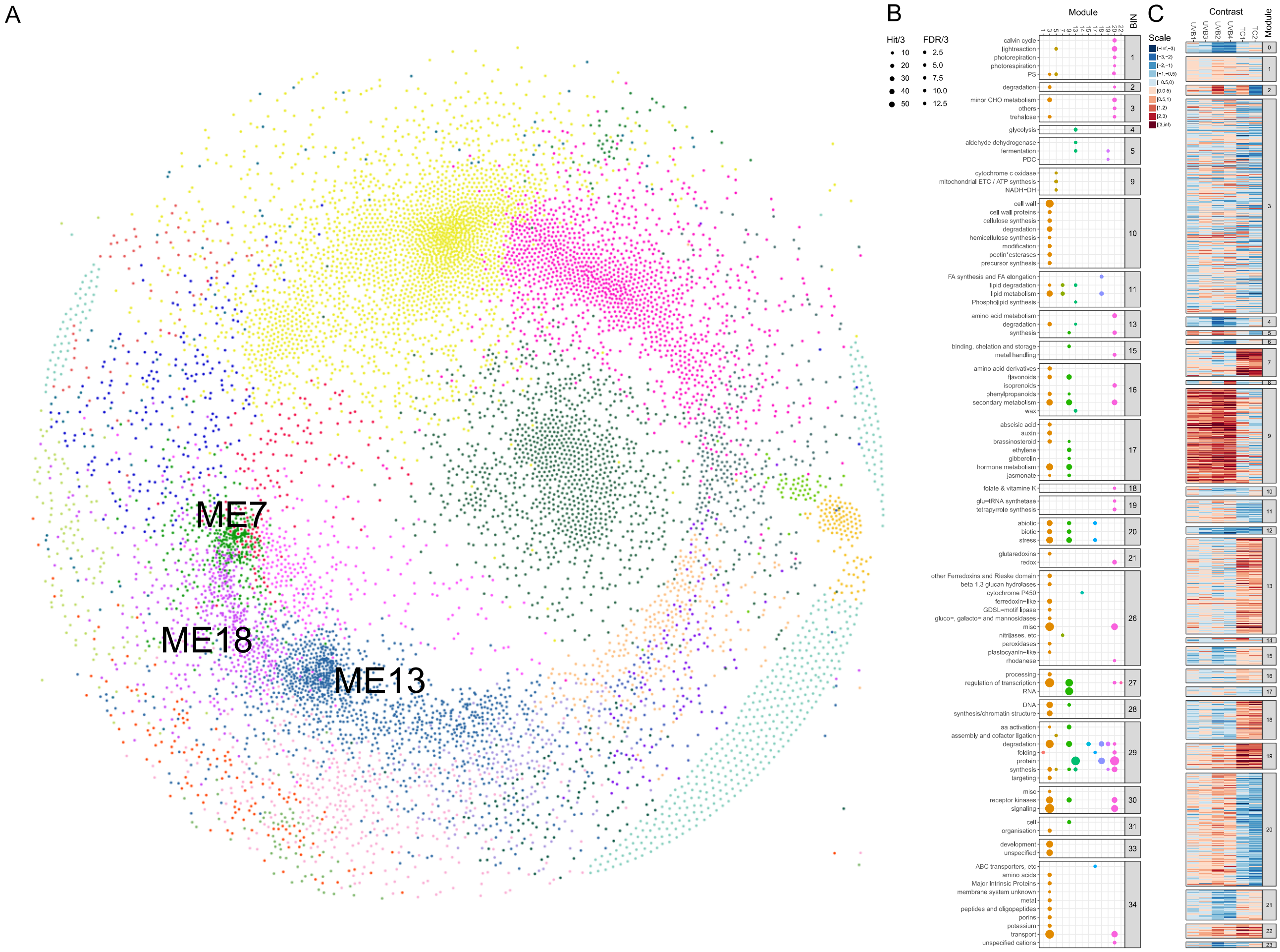

Supplement: Supplementary file 9 [file Image_1.pdf]
