# Supplementary material for: Tissue-Specific Floral Transcriptome Analysis of the Sexually Deceptive Orchid Chiloglottis trapeziformis Provides Insights into the Biosynthesis and Regulation of Its Unique UV-B Dependent Floral Volatile, Chiloglottone 1
Source: Front Plant Sci. 2017 Jul 19;8:1260. doi: 10.3389/fpls.2017.01260 (PMC5515871; doi:10.3389/fpls.2017.01260)
Supplement: Supplementary file 10 [file Table_1.docx]

**Supplementary Table 1.** List of transcripts highlighted in this study. All associated information (Cluster ID, symbol, meta-annotation, differential expression contrast, and metabolic pathway) for highlighted transcripts in the (A) UV-B treatment and (B) tissue-specific sections are listed. ^+^ denotes TRAPID meta-annotation for each transcript. Only the top most meta-annotation assignment for each transcript is listed, beginning with ‘Full Length’ followed by ‘Quasi Full Length’, ‘Partial’, and ‘No information’. GLV, Green leaf volatile; ABA, Abscisic acid, JA, Jasmonic acid; Phe/Fla, Phenylpropanoid/Flavonoid; FA, Fatty acid.

**A**

| Cluster ID | Symbol | Meta-annotation^+^ | Contrast | Pathway |
| --- | --- | --- | --- | --- |
| CHILtrapF-81207.0 | HY5 HOMOLOG | Full Length | UV-B | UVR8-signaling |
| CHILtrapF-88088.0 | HY5 | Full Length | UV-B | UVR8-signaling |
| CHILtrapF-87699.0 | RUP2 | Full Length | UV-B | UVR8-signaling |
| CHILtrapF-109614.1 | SPA3/4 | Full Length | UV-B | UV-B-response |
| CHILtrapF-73004.0 | ELIP1 | Quasi Full Length | UV-B | UV-B-response |
| CHILtrapF-108513.0 | NCED3a | Full Length | UV-B | ABA |
| CHILtrapF-96073.0 | NCED3b | Quasi Full Length | UV-B | ABA |
| CHILtrapF-109170.0 | bLCY1/eCYC | Full Length | UV-B | Carotenoid |
| CHILtrapF-88422.1 | CRISTOa | Full Length | UV-B | Carotenoid |
| CHILtrapF-76911.1 | CRISTOb | Full Length | UV-B | Carotenoid |
| CHILtrapF-112245.2 | CRISTOc | Full Length | UV-B | Carotenoid |
| CHILtrapF-70778.1 | VDEa | Full Length | UV-B | Carotenoid |
| CHILtrapF-70778.0 | VDEb | Quasi Full Length | UV-B | Carotenoid |
| CHILtrapF-90118.0 | βOHase1/2 | Full Length | UV-B | Carotenoid |
| CHILtrapF-107392.0 | εOHase | Full Length | UV-B | Carotenoid |
| CHILtrapF-105492.0 | 13-LOXa | Full Length | UV-B | GLV |
| CHILtrapF-87688.0 | 13-LOXb | Full Length | UV-B | GLV |
| CHILtrapF-106113.0 | 9-LOXa | Full Length | UV-B | GLV |
| CHILtrapF-111903.0 | 9-LOXb | Quasi Full Length | UV-B | GLV |
| CHILtrapF-77254.0 | OAC | Full Length | UV-B | JA |
| CHILtrapF-105368.0 | OPRa | Partial | UV-B | JA |
| CHILtrapF-105368.1 | OPRb | Quasi Full Length | UV-B | JA |
| CHILtrapF-68405.0 | ADT | Full Length | UV-B | Phe/Fla |
| CHILtrapF-75039.26 | CHS | Full Length | UV-B | Phe/Fla |
| CHILtrapF-94341.0 | DAHPSa | Quasi Full Length | UV-B | Phe/Fla |
| CHILtrapF-86596.0 | DAHPSb | Full Length | UV-B | Phe/Fla |
| CHILtrapF-82878.0 | DFR | Full Length | UV-B | Phe/Fla |
| CHILtrapF-87880.0 | F3'Ha | Full Length | UV-B | Phe/Fla |
| CHILtrapF-103275.0 | F3'Hb | Full Length | UV-B | Phe/Fla |
| CHILtrapF-8088.9 | F3'Hc | Quasi Full Length | UV-B | Phe/Fla |
| CHILtrapF-109638.0 | F3'Hd | Full Length | UV-B | Phe/Fla |
| CHILtrapF-77032.0 | PALa | Quasi Full Length | UV-B | Phe/Fla |
| CHILtrapF-90578.0 | PALb | Quasi Full Length | UV-B | Phe/Fla |
| CHILtrapF-81175.0 | TPS1 | Full Length | UV-B | Terpene |

**B**

| Cluster ID | Symbol | Meta-annotation* | Contrast | Pathway |
| --- | --- | --- | --- | --- |
| CHILtrapF-100692.0 | AAD1 | Full Length | TC | FA |
| CHILtrapF-105042.1 | FAB2L1 | Full Length | TC | FA |
| CHILtrapF-105042.0 | FAB2L2 | Full Length | TC | FA |
| CHILtrapF-96900.1 | FATB2 | Full Length | TC | FA |
| CHILtrapF-92387.0 | FATB3 | Full Length | TC | FA |
| CHILtrapF-89251.0 | KAR-L1 | Full Length | TC | FA |
| CHILtrapF-92359.4 | KAR-L2 | Full Length | TC | FA |
| CHILtrapF-92359.3 | KAR-L3 | Full Length | TC | FA |
| CHILtrapF-87481.0 | KASI-1 | Full Length | TC | FA |
| CHILtrapF-109537.0 | KASI-2 | Full Length | TC | FA |
| CHILtrapF-84874.3 | KASIII | Full Length | TC | FA |
| CHILtrapF-87251.0 | KAT2 | Full Length | TC | FA |
| CHILtrapF-72497.0 | KAT2L | Full Length | TC | FA |
| CHILtrapF-114555.0 | LACS1/4 | Full Length | TC | FA |
| CHILtrapF-90258.0 | LACS2/3 | Full Length | TC | FA |
| CHILtrapF-95937.0 | LACS6/7 | Full Length | TC | FA |
| CHILtrapF-86517.0 | LACS8/9 | Full Length | TC | FA |
| CHILtrapF-92359.5 | mtKAR | Full Length | TC | FA |
| CHILtrapF-94841.0 | ACX2/3 | Full Length | TC | FA |
| CHILtrapF-87380.0 | ACX3/6 | Full Length | TC | FA |
| CHILtrapF-80808.0 | ACX4 | Full Length | TC | FA |
| CHILtrapF-79124.0 | ACX1/5 | Full Length | TC | FA |
| CHILtrapF-83623.0 | MFP1 | Full Length | TC | FA |
| CHILtrapF-72378.0 | MFP3 | Full Length | TC | FA |
| CHILtrapF-92970.0 | MFP4 | Full Length | TC | FA |
| CHILtrapF-83214.0 | MFP5 | Full Length | TC | FA |
| CHILtrapF-93572.1 | MFP6 | Full Length | TC | FA |
